# Supplementary material for: Valine–Niclosamide for Treatment of Androgen Receptor Splice Variant-Positive Hepatocellular Carcinoma
Source: Cancers (Basel). 2025 Jul 31;17(15):2535. doi: 10.3390/cancers17152535 (PMC12346198; doi:10.3390/cancers17152535)
Supplement: Supplementary file 1 [file cancers-17-02535-s001.zip › Original_Uncropped_Western_Blot_Images.pdf]

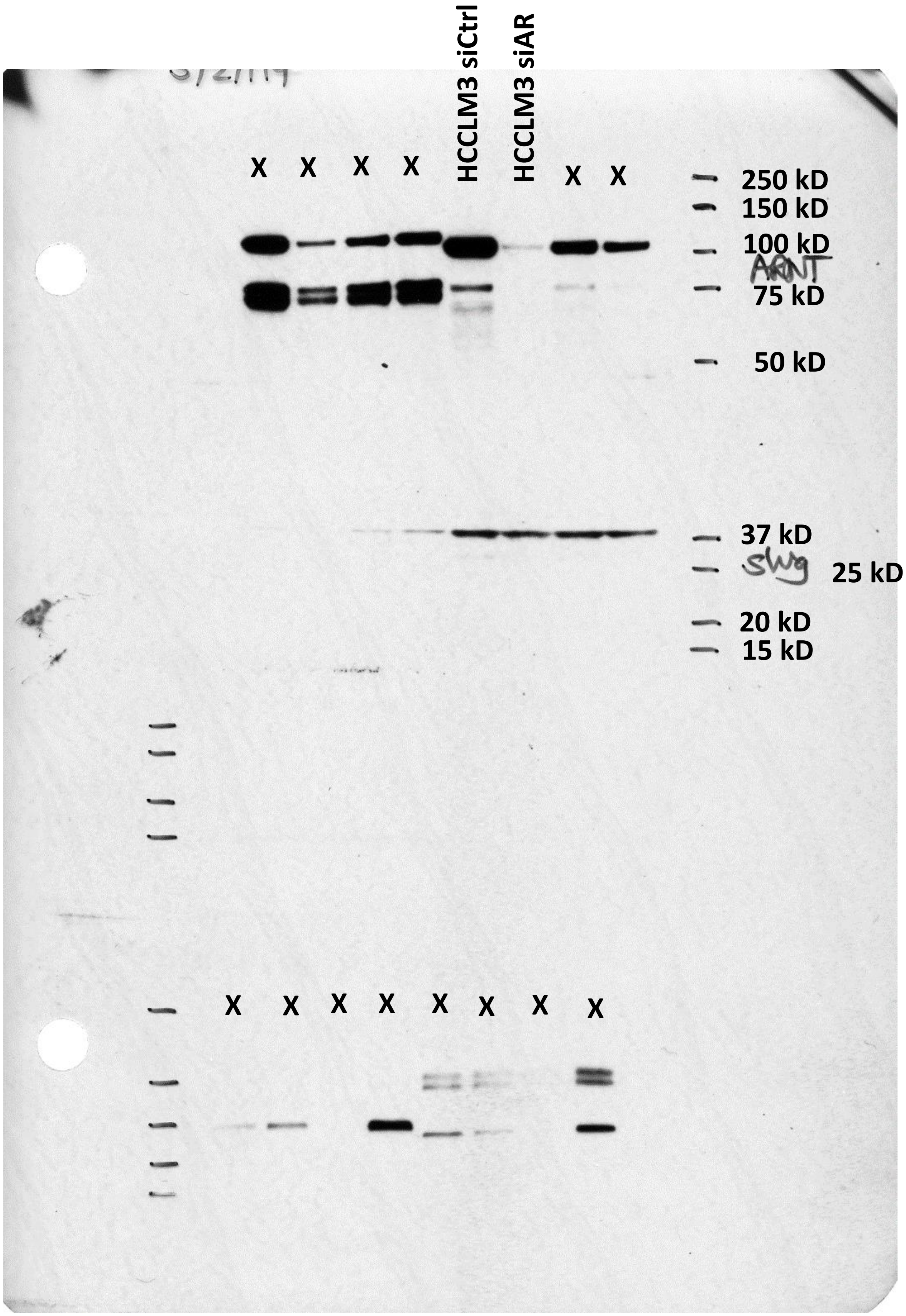

Uncropped Western Blot for Figure 1B HCCLM3 AR-NT. AR status of siAR transfected cells was confirmed by western blot with anti-AR-NT (CS#5153, Cell Signaling).

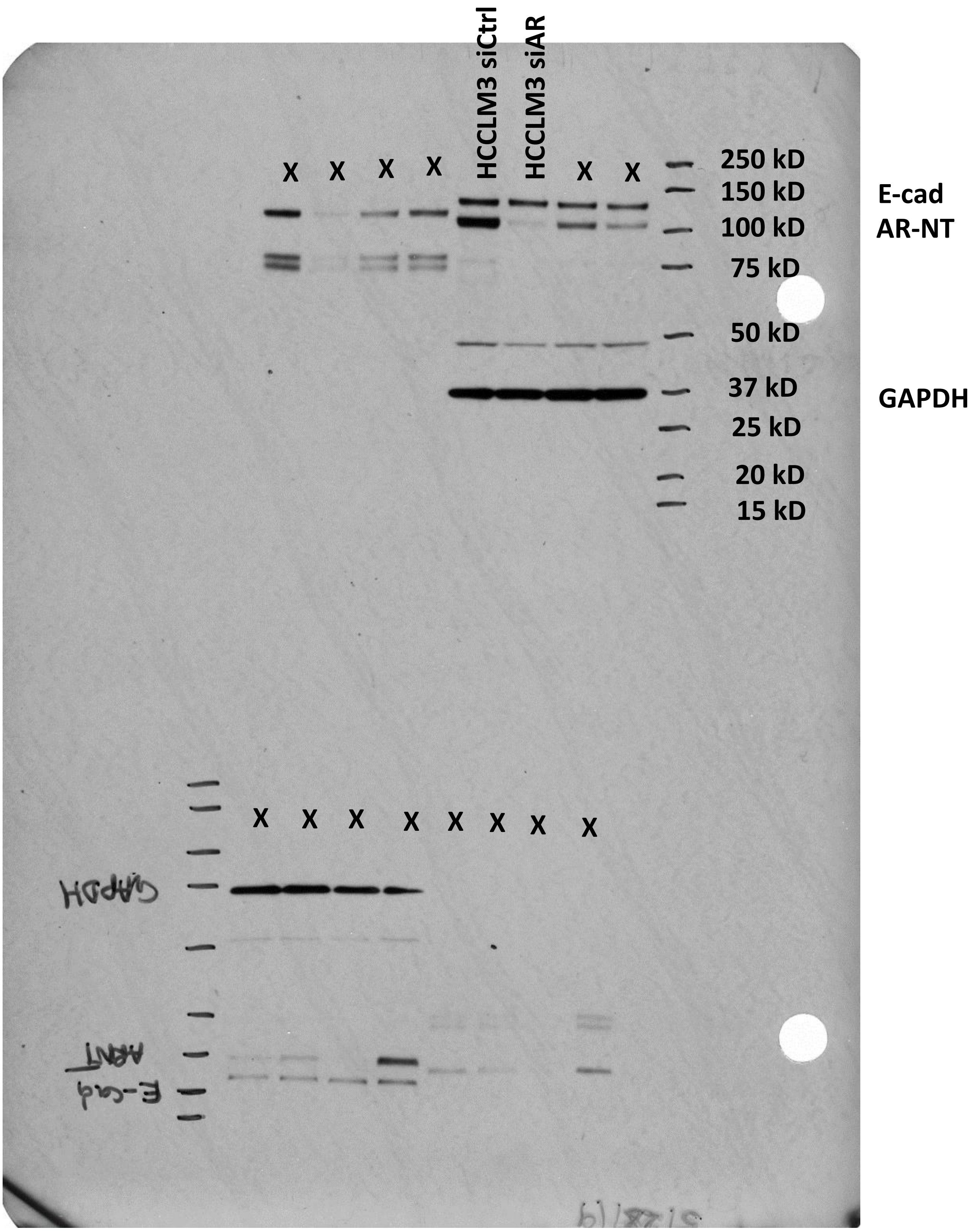

Uncropped Western Blot for Figure 1B HCCLM3 GAPDH. AR status of siAR HCCLM3 transfected cells was confirmed by western blot with GAPDH (CS#5174, Cell Signaling).

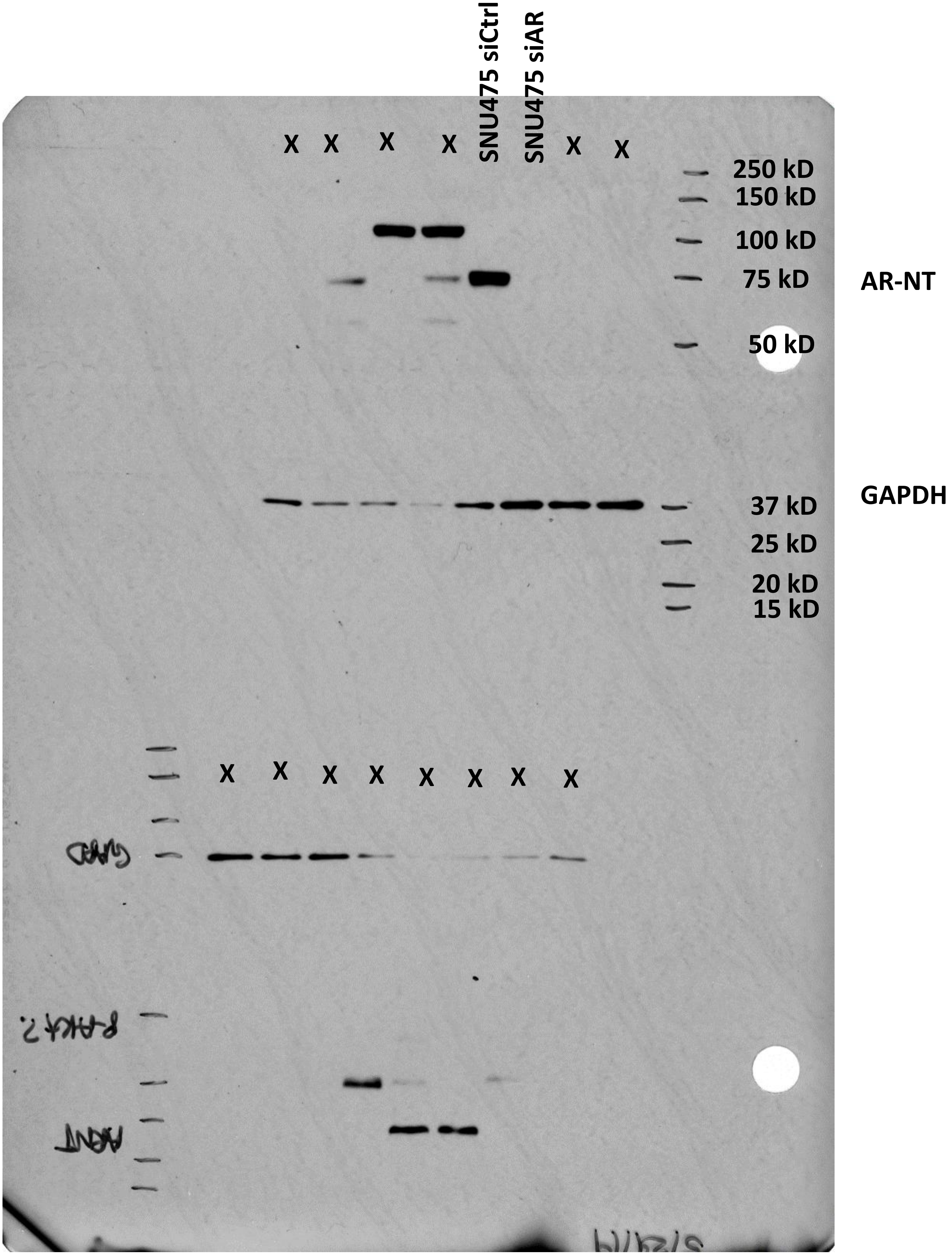

Uncropped Western Blot for Figure 1B SNU475 AR-NT and GAPDH. AR status of siAR SNU475 transfected cells was confirmed by western blot with anti-AR-NT (CS#5153, Cell Signaling) and GAPDH (CS#5174, Cell Signaling).
