# Supplementary material for: Valine–Niclosamide for Treatment of Androgen Receptor Splice Variant-Positive Hepatocellular Carcinoma
Source: Cancers (Basel). 2025 Jul 31;17(15):2535. doi: 10.3390/cancers17152535 (PMC12346198; doi:10.3390/cancers17152535)
Supplement: Supplementary file 1 [file cancers-17-02535-s001.zip › Valine-Niclosamide_Manuscript_Supplemental_Material_Draft.pdf]

```
WT
ATGCAACTCCTTCAGCAACAGCAGCAGGAAGCAGTATCCGAAGGCAGCAGCAGCGGGAGAGCGAGGGAGGCCTCGGGGGCTCCCACTTCCTCCAAGGACAATTACTTAGGGGGCACTTCGACCATTTCTGACAACGCCAAGGAGTTGTG
T
                                     (-70 nts)
#35-1 ATGCAACTCCTTCAGCAACA-------------------------------------------------------
TCCAAGGACAATTACTTAGGGGGCACTTCGACCATTTCTGACGACGCCAAGGAGTTGTGT
                                                                    (-116 nts)
#35-2 ATGCAACTCCTTCAGCAAC-------------------------------------------------------------------------------------------
GCCAAGGAGTTGTGT
                                               (-68 nts)                                   (-2
nts)
#35-3 ATGCAACTCCTTCAGCAACAGC-----------------------------------------------------TCCAAGGACAATTACTTAGGGGGCACTTCGACCAT-
CTGACAACGCCAAGGAGTTGTGT

      aa196
WT   MQLLQQQQQEAVSEGSSSGRAREASGAPTSSKDNYLGGTSTISDNAKELCKAVSVSMGLGVEALEHLSPGEQLRGDCMYAPLLGVPPAVRPTPCAPLAEC
#35  MQLLQQHPRTIT*
     MQLLQQRQGVV*
     MQLLQQQLQGQLLRGHFDHLTTPRSCVRQCRCPWAWVWRRWSI*


WT
ATGCAACTCCTTCAGCAACAGCAGCAGGAAGCAGTATCCGAAGGCAGCAGCAGCGGGAGAGCGAGGGAGGCCTCGGGGGCTCCCACTTCCTCCAAGGACAATTACTTAGGGGGCACTTCGACCATTTCTGACAACGCCAAGGAGTTGTG
T
                                                   (-69 nts)                               (-19 nts)
#38-1 ATGCAACTCCTTCAGCAACAG------------------------------------------------------TCCAAGGACAATTACTTAG---------------
TGACAACGCCAAGGAGTTGTGT
                          (-3 nts)                                                    (-38 nts)
#38-2 ATGCAACTCCTTCAGCAACAGCAG---GAAGCAGTATCCGAAGGCAGCAGCAGCGGGAGAGCGAGGGAGGCCTCGGGGGCTCCCACTTCCT------------------------------
GACAACGCCAAGGAGTTGTGT

       aa197
WT   MQLLQQQQQEAVSEGSSSGRAREASGAPTSSKDNYLGGTSTISDNAKELCKAVSVSMGLGVEALEHLSPGEQLRGDCMYAPLLGVPPAVRPTPCAPLAEC
#38  MQLLQQQSKDNYLVTTPRSCVRQCRCPWAWVWRRWSI*
     MQLLQQQQEAVSEGSSSGRAREASGAPTS*


WT
ATGCAACTCCTTCAGCAACAGCAGCAGGAAGCAGTATCCGAAGGCAGCAGCAGCGGGAGAGCGAGGGAGGCCTCGGGGGCTCCCACTTCCTCCAAGGACAATTACTTAGGGGGCACTTCGACCATTTCTGACAACGCCAAGGAGTTGTG
T
                                    (-116 nts)
#62-1 ATGCAACTCCTTCAGCAAC-------------------------------------------------------------------------------------------
GCCAAGGAGTTGTGT
                   (-4 nts)                                                              (-36 nts)
#62-2 ATGCAACTCCTTCAGCAA----AGCAGGAAGCAGTATCCGAAGGCAGCAGCAGCGGGAGAGCGAGGGAGGCCTCGGGGGCTCCCACTTCCTC----------------------------
TGACAACGCCAAGGAGTTGTGT

WT    ATGCAACTCCTTCAGCAACAGCAGCAGGAAGCAGTATCCGAAGGCAGCAGCAGCGGGAGAGCGAGGGAGGCCTCGGGGGCTCCCACTTCCTCCAAGGACAATTACTTAGGGGGCACTTCGACCATTT--
CTGACAACGCCAAGGAGTTGTG
#62-3 ATGCAACTCCTTCAGCAACAG-
CTCCAAGGACAATTACTTAGGGGGCACTTCGACCATTTAGCAGGAAGCAGTATCCGAAGGCAGCAGCAGCGGGAGAGCGAGGGAGGCCTCGGGGGCTCCCACTTCCTCTGACAACGCCAAGGTGTTGTGT
                    (-1 nt)                         inversion                                     (+2
nts)

      aa196
WT   MQLLQQQQQEAVSEGSSSGRAREASGAPTSSKDNYLGGTSTISDNAKELCKAVSVSMGLGVEALEHLSPGEQLRGDCMYAPLLGVPPAVRPTPCAPLAEC
#62  MQLLQQRQGVV*
     MQLLQQSRKQYPKAAAAGERGRPRGLPLPLTTPRSCVRQCRCPWAWVWRRWSI*
     MQLLQQQLQGQLLRGHFDHLAGSSIRRQQQRESEGGLGGSHFL*


WT
ATGCAACTCCTTCAGCAACAGCAGCAGGAAGCAGTATCCGAAGGCAGCAGCAGCGGGAGAGCGAGGGAGGCCTCGGGGGCTCCCACTTCCTCCAAGGACAATTACTTAGGGGGCACTTCGACCATTTCTGACAACGCCAAGGAGTTGTG
T
                                                         (-109 nts)
#65-1 ATGCAACTCCTTCAGCAAC-------------------------------------------------------------------------------------
TGACAACGCCAAGGAGTTGTGT
                                                   (-107 nts)
#65-2 ATGCAACTCCTTCAGCAACAGC------------------------------------------------------------------------------------
GACAACGCCAAGGAGTTGTGT
                      (-2 nts)                                                              (-36 nts)
#65-3 ATGCAACTCCTTCAGCAACA--AGCAGGAAGCAGTATCCGAAGGCAGCAGCAGCGGGAGAGCGAGGGAGGCCTCGGGGGCTCCCACTTCCTC----------------------------
TGACAACGCCAAGGAGTTGTGT

      aa196
WT   MQLLQQQQQEAVSEGSSSGRAREASGAPTSSKDNYLGGTSTISDNAKELCKAVSVSMGLGVEALEHLSPGEQLRGDCMYAPLLGVPPAVRPTPCAPLAEC
#65  MQLLQQLTTPRSCVRQCRCPWAWVWRRWSI*
     MQLLQQQRQRQGVV*
     MQLLQQQAGSSIRRQQQRESEGGLGGSHFL*
```

Figure S1. Sequencing of HCCLM3 CRISPR AR KO clones. DNA and amino acid sequences of CRISPR AR KO HCCLM3 clones (#35, #38, #62, and #65) compared to HCCLM3 wild type demonstrating pre-mature stop codons consistent with AR gene knockout. The number of deleted nucleotides is presented in parenthesis above the edited DNA strand. Amino acids created by frame shift are indicated by red font. Highlighted in yellow is the last amino acid of the wild type N terminus. Yellow and green segments of amino acids indicate the inversion and underlined bold CT dinucleotide represents an insertion.

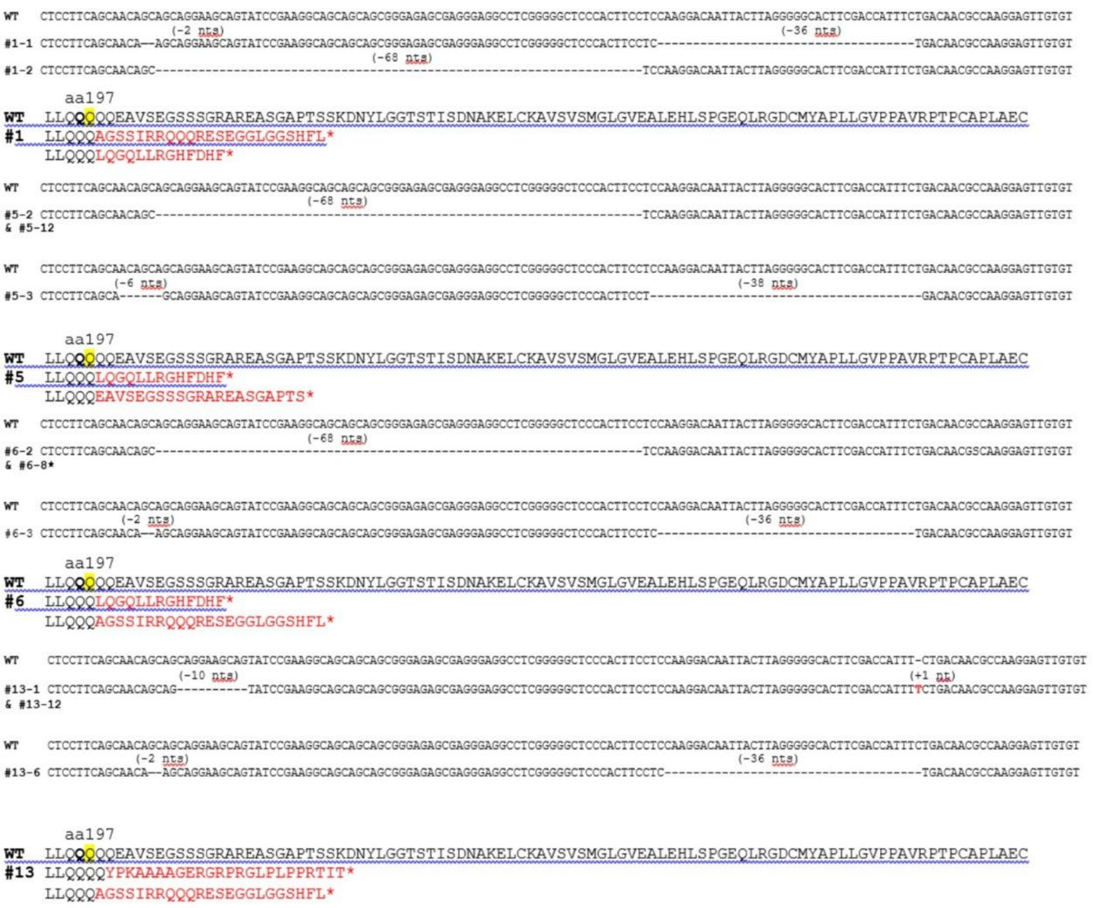

Figure S2. Sequencing of SNU475 CRISPR AR KO clones. DNA and amino acid sequences of CRISPR AR KO SNU475 clones (#1, #5, #6, and #13) compared to SNU475 wild type demonstrating pre-mature stop codons consistent with AR gene knockout. For detailed legend, see the legend for Supplemental Figure 1.

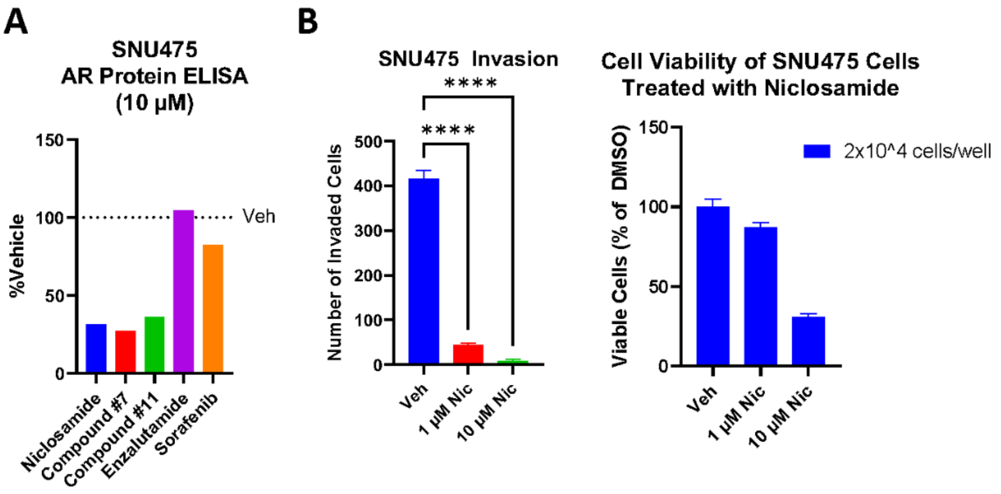

Figure S3. Biological activity of niclosamide on SNU475 cells. (**A**) SNU475 cells were treated with 10 µM of each compound for 24 hours before analysis by AR ELISA. (**B**) (Left) Cellular invasion assay was performed on SNU475 cells plated at 2x10^4 cells/well treated with 1 and 10 µM niclosamide for 48 hrs. Mean ± SD One way ANOVA with Dunnett's multiple comparisons test. ****, $P<0.0001$ versus niclosamide treated cells. (Right) Cell viability was performed under the same conditions as invasion assay, 2x10^4 cells/well treated with either 1 or 10 µM niclosamide for 48 hours, Mean ± SD.

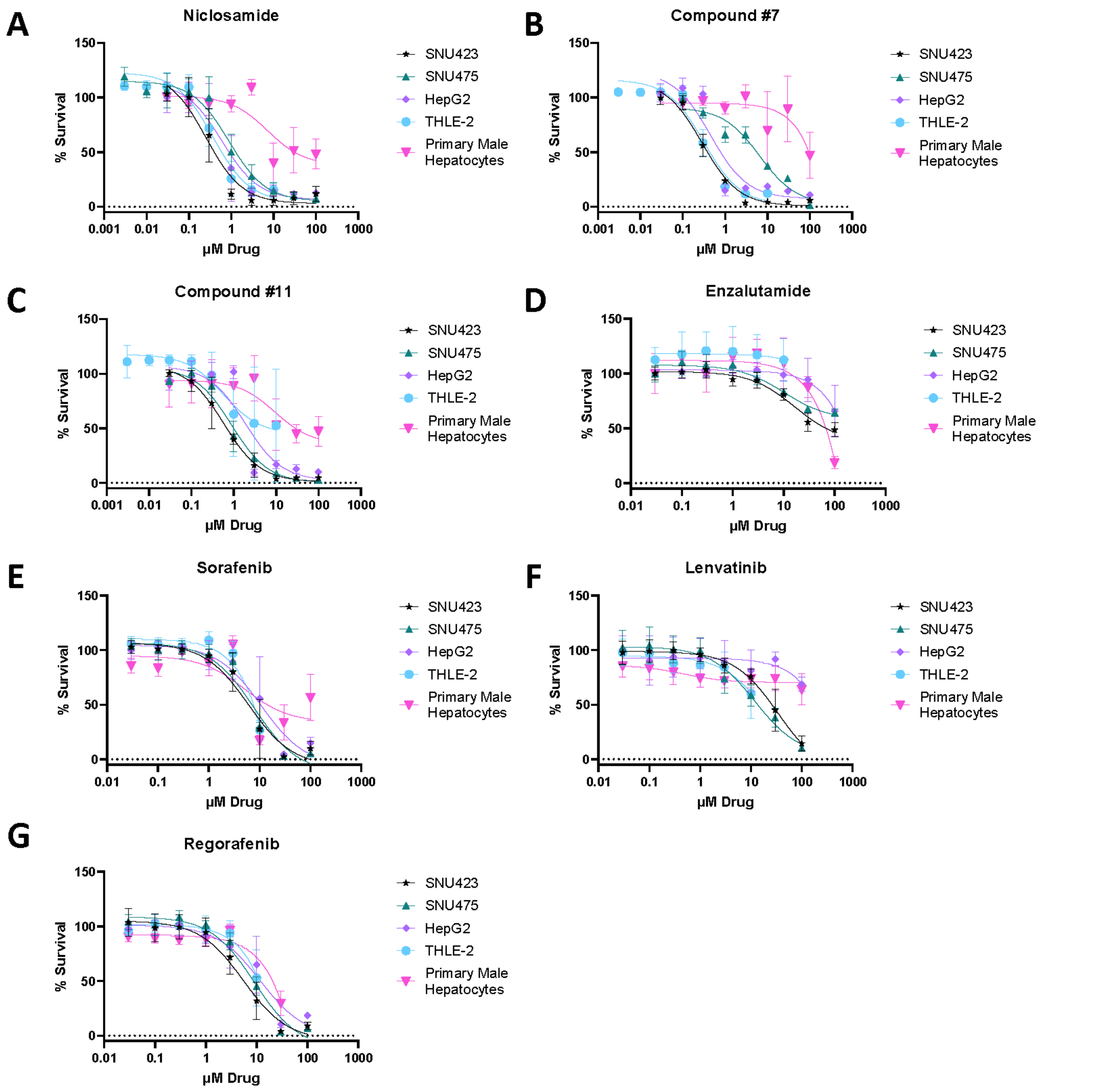

Figure S4. Cytotoxicity dose response for niclosamide analogs and standard of care drugs. (**A**) Cytotoxicity dose response of niclosamide against HCC cell lines SNU423, SNU475, HepG2, normal immortalized liver THLE-2, and primary male hepatocytes. (**B**) Cytotoxicity dose response of compound #7. (**C**) Cytotoxicity dose response of compound #11 against HCC cell lines SNU423, SNU475, HepG2, normal immortalized liver THLE-2, and primary male hepatocytes. (**D**) Cytotoxicity dose response of enzalutamide, an antiandrogen drug, against HCC cell lines SNU423, SNU475, HepG2, normal immortalized liver THLE-2, and primary male hepatocytes. (**E**) Cytotoxicity dose response of sorafenib, an HCC standard of care, against HCC cell lines SNU423, SNU475, HepG2, normal immortalized liver THLE-2, and primary male hepatocytes. (**F**) Cytotoxicity dose response of lenvatinib, an HCC standard of care, against HCC cell lines SNU423, SNU475, HepG2, normal immortalized liver THLE-2, and primary male hepatocytes. (**G**) Cytotoxicity dose response of regorafenib, an HCC standard of care, against HCC cell lines SNU423, SNU475, HepG2, normal immortalized liver THLE-2, and primary male hepatocytes. n=6-18, representing at least three separate biological replicates occurring across at least three passages.

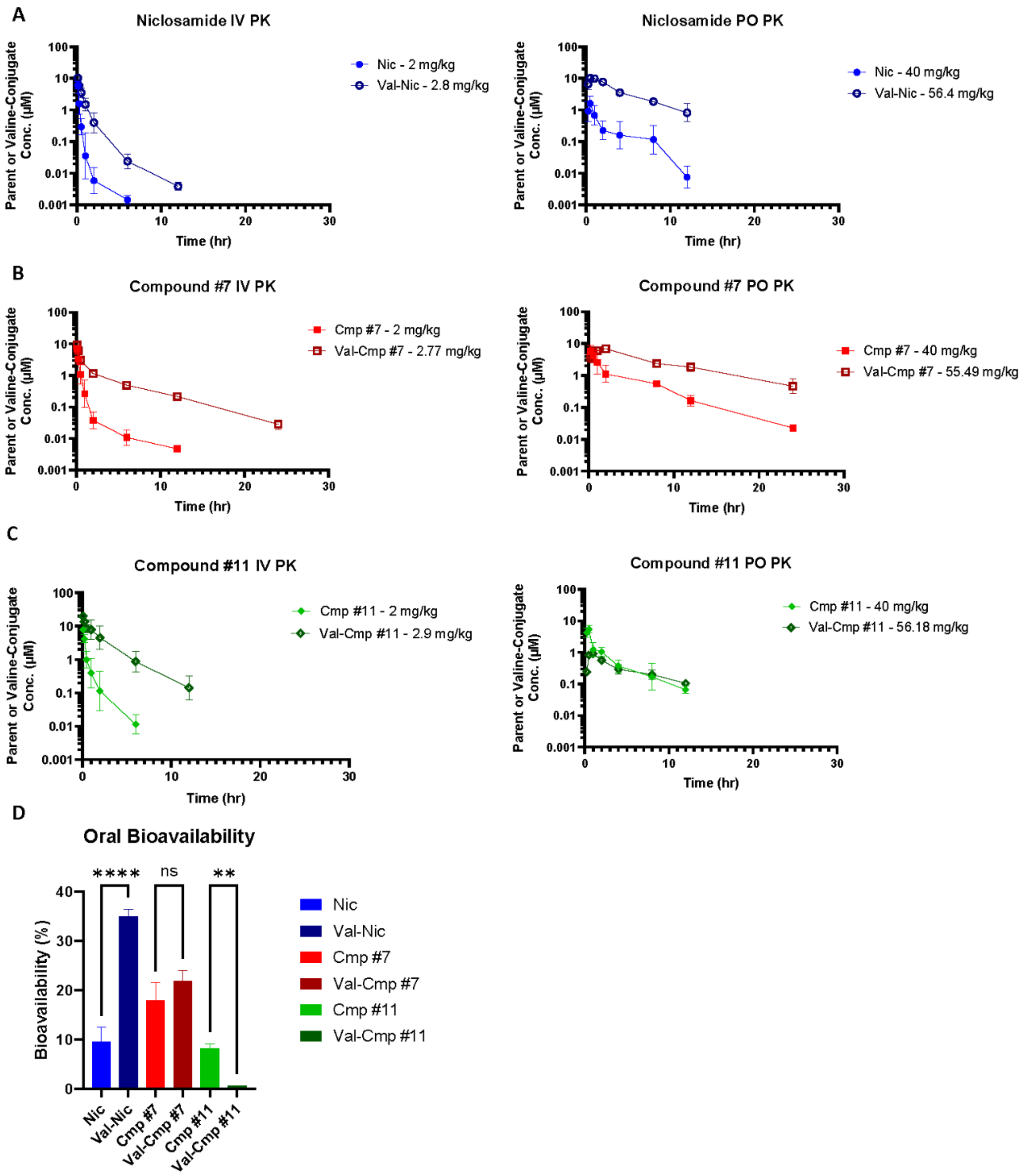

Figure S5. Concentration vs time curves and bioavailability of niclosamide analogs and valine-conjugates in mouse. (**A**) Single dose PK of niclosamide at 2 mg/kg IV and 40 mg/kg PO were niclosamide was measured and single dose PK of valine niclosamide at molar equivalent doses where valine-niclosamide was measured is shown. (**B**) Single dose PK of compound #7 at 2 mg/kg IV and 40 mg/kg PO and single dose PK of valine-compound #7 at molar equivalent doses shown. (**C**) Single dose PK of compound #11 at 2 mg/kg IV and 40 mg/kg PO and single dose PK of valine-compound #11 at molar equivalent doses shown. (**D**) Bioavailability of all niclosamide analogs and valine conjugates was determined. Mean±SD, One way ANOVA with Šidák's multiple comparisons test. ns, P>0.05; **, P<0.01; ****, P<0.0001 versus respective parent compound.

Table S1. Pharmacokinetic parameters from single IV or single PO doses of niclosamide amino-acid conjugates and analogs.[1]

			**AUC_{all} (hr*μmol/L)**		**IV: Cl_{obs} (L/hr) PO: Cl_F_{obs} (L/hr)**		**C_{max} (μmol/L)**		**HL_Lambda_z (hr)**		**T_{max} (hr)**	
**Dose**	**Route**	**Analyte**	**Geo Mean**	**Geo CV%**	**Geo Mean**	**Geo CV%**	**Geo Mean**	**Geo CV%**	**Geo Mean**	**Geo CV%**	**Geo Mean**	**Geo CV%**
2 mg/kg	IV	Nic	1.79	37.47	0.08	28.44	6.03	26.71	1.33	68.33	0.08	0.00
2 mg/kg EQV	IV	Val-Nic	7.52	34.60	0.02	35.28	11.20	8.31	1.54	13.02	0.08	0.00
2 mg/kg	IV	Cmp #7	3.70	20.93	0.04	22.88	6.98	4.19	4.85	10.04	0.08	0.00
2 mg/kg EQV	IV	Val-Cmp #7	13.46	5.07	0.01	2.74	10.08	11.69	4.20	3.78	0.08	0.00
2 mg/kg	IV	Cmp #11	5.06	27.11	0.03	29.72	11.31	5.36	0.96	21.20	0.08	0.00
2 mg/kg EQV	IV	Val-Cmp #11	36.06	55.80	0.004	58.38	20.17	15.32	1.94	10.42	0.39	20.01
40 mg/kg	PO	Nic	3.25	37.85	0.78	55.85	1.65	56.00	2.74	75.99	0.42	34.64
40 mg/kg EQV	PO	Val-Nic	48.57	8.21	0.05	10.40	11.15	11.00	4.11	58.21	0.67	43.30
40 mg/kg	PO	Cmp #7	12.97	8.04	0.21	8.08	5.79	18.79	3.58	3.25	0.25	0.00
40 mg/kg EQV	PO	Val-Cmp #7	62.09	1.21	0.05	5.95	7.71	0.94	6.46	14.37	1.26	35.84
40 mg/kg	PO	Cmp #11	7.98	14.35	0.35	10.15	6.08	16.38	2.68	34.97	0.42	34.64
40 mg/kg EQV	PO	Val-Cmp #11	4.17	15.84	0.59	8.82	0.99	3.92	5.77	32.48	1.00	0.00

[1] Pharmacokinetic parameters were determined by non-compartmental analysis.

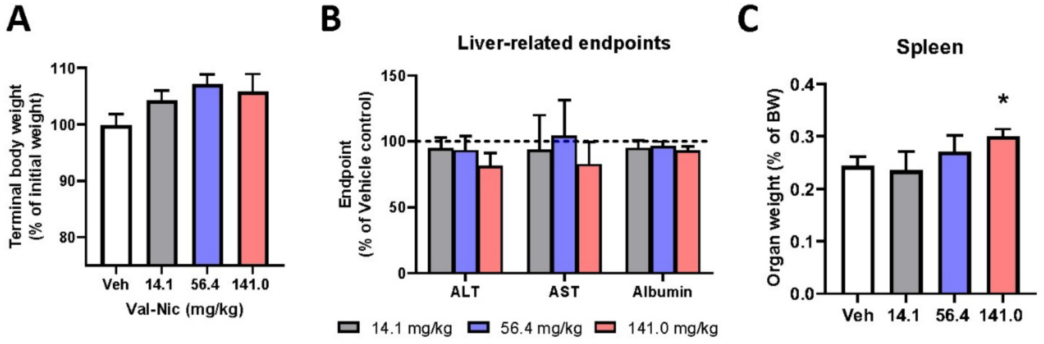

Figure S6. Valine-niclosamide tolerance study. (**A**) Terminal body weights of male C57BL/6J mice following 2 weeks of daily oral administration of 14.1, 56.4, or 141.0 mg/kg doses of valine-niclosamide (molar equivalents of 10, 40, and 100 mg/kg niclosamide). (**B**) Liver enzyme panel comprised of alanine aminotransferase (ALT), aspartate aminotransferase (AST), and albumin completed post-study. (**C**) Spleen weights as a percentage of body weight with the only significant difference being an increase at the highest dose level. Mean ±SD, One way ANOVA with Dunnett's multiple comparisons test. *, $P<0.05$ versus vehicle.

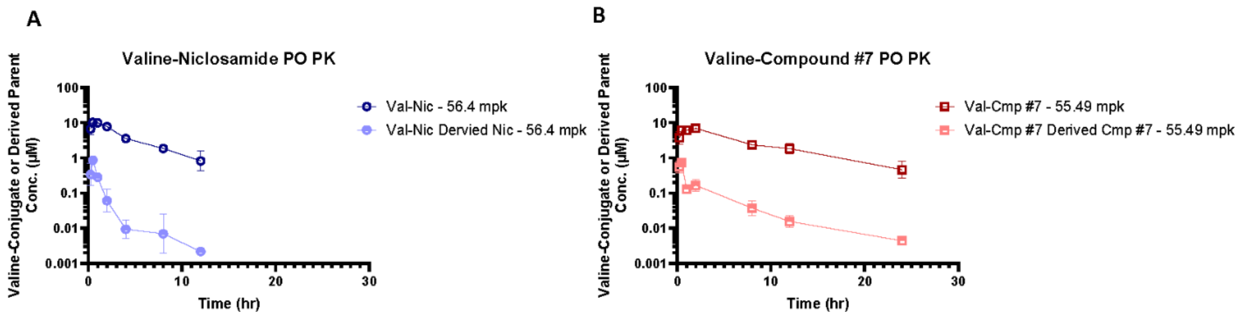

Figure S7. Concentration vs time curves of parent drug and valine-conjugate from oral doses of valine-conjugates in mouse. (**A**) Single dose PK of valine-niclosamide at 40 mg/kg PO molar equivalent with measured prodrug and parent (niclosamide) derived from prodrug concentrations. (**B**) Single dose PK of valine-compound #7 at 40 mg/kg PO molar equivalent with measured prodrug and parent (compound #7) derived from prodrug concentrations.

Table S2. Pharmacokinetic parameters from single PO doses of niclosamide amino-acid conjugates measuring parent drug.[1]

			AUC_{all} (hr*µmol/L)		IV: Cl_{obs} (L/hr) PO: Cl_F_{obs} (L/hr)		C_{max} (µmol/L)		HL_Lambda_z (hr)		T_{max} (hr)	
**Dose**	**Route**	**Analyte**	**Geo Mean**	**Geo CV%**	**Geo Mean**	**Geo CV%**	**Geo Mean**	**Geo CV%**	**Geo Mean**	**Geo CV%**	**Geo Mean**	**Geo CV%**
40 mg/kg EQV	PO	Nic	0.80	22.99	3.45	18.79	0.87	18.69	3.52	72.71	0.50	0.00
40 mg/kg EQV	PO	Cmp #7	1.31	3.47	2.33	4.19	0.85	4.03	4.83	6.56	0.40	17.51

[1] Pharmacokinetic parameters were determined by non-compartmental analysis.

$$\text{Amount of metabolite formed} = \int_0^T k_{met} \cdot A_{prodrug}(t)\, dt$$

Substituting $A_{prodrug}(t)$:

$$\text{Amount of metabolite formed} = \int_0^T k_{met} \cdot A_{prodrug,0} \cdot e^{-(k_{el}+k_{met})\cdot t}\, dt$$

This evaluates to:

$$\text{Amount of metabolite formed} = \frac{A_{prodrug,0} \cdot k_{met}}{k_{el} + k_{met}} \cdot \left[1 - e^{-(k_{el}+k_{met})\cdot T}\right]$$

Where:

- T: Time at which you want the total metabolite amount.
- $A_{prodrug,0}$: Initial dose of the prodrug.

Figure S8. Mathematical equations for the calculation of the amount of metabolite formed over time. K_{met} - First-Order Rate of Metabolite Formation , K_{el} - First order elimination rate constant of the prodrug

A

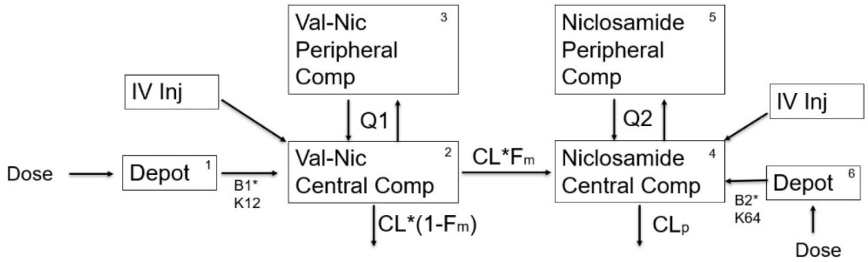

B

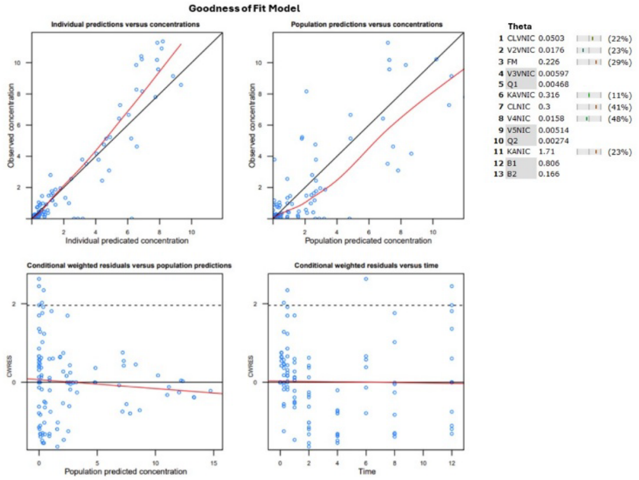

C

Parameter	Source	Estimate
Clearance of Valine-Niclosamide (Cl_{VN})	PopPK Model	0.05 L/hr
Volume of Central Valine- Niclosamide Compartment (V_2)	PopPK Model	0.0176 L
First-Order Rate of Elimination (K_{el})	$Cl_{VN} \div V_2$	2.84 hr^{-1}
Fraction Metabolized (F_m)	PopPK Model	0.226
First-Order Rate of Metabolite Formation (K_{met})	$F_m \times K_{el}$	0.641 hr^{-1}
Half-Life of the Conversion Process	$\ln(2)/K_{met}$	1.08 hrs
Amount of Metabolite Formed in 5 Half-Lives	*	0.525 µmoles
Rate of Metabolite Formation	0.525 / 5.4	0.1 µmoles/hr

Figure S9. Conversion of valine-conjugated niclosamide to niclosamide. (A) Flow chart of pharmacokinetic modeling scheme integrating single IV and PO doses of both niclosamide and valine-conjugated niclosamide. B1, bioavailability of valine niclosamide; K12, first order absorption rate constant of valine niclosamide; Q1 and Q2, inter-compartmental clearance; CL, clearance of valine niclosamide; Fm, fraction of valine-niclosamide metabolized to niclosamide; CLp, clearance of the niclosamide; B2, bioavailability of niclosamide ; K64, first order absorption rate constant of niclosamide. (B) Goodness of fit plots and model estimated parameters of the PopPK model. Individual predicted versus observed concentration (top left panel); Population predicted versus observed concentration (top right panel); Conditional weighted residuals versus population predicted concentration (bottom left panel); Conditional weighted residuals versus time (bottom right panel); Theta- Model estimated population parameters. (C) Table outline of the model estimated parameters and equations used to derive the rate of metabolite formation. * is calculated from pharmacokinetic equation for metabolite formation as shown in Figure S8.

A

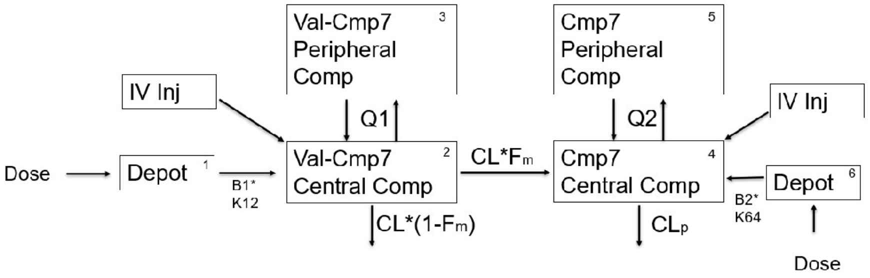

B

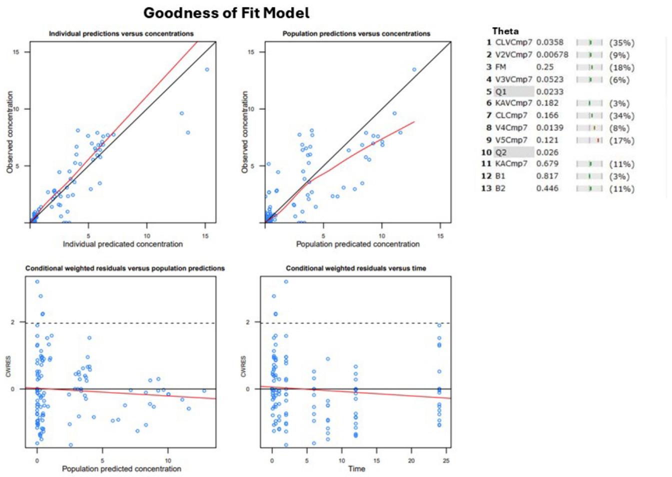

C

Parameter	Source	Estimate
Clearance of Valine-Compound #7 (Cl_{V7})	PopPK Model	0.0358 L/hr
Volume of Central Valine-Compound #7 Compartment (V_2)	PopPK Model	0.00678 L
First-Order Rate of Elimination (K_{el})	$Cl_{VN} \div V_2$	5.28 hr^{-1}
Fraction Metabolized (F_m)	PopPK Model	0.25
First-Order Rate of Metabolite Formation (K_{met})	$F_m \times K_{el}$	1.32 hr^{-1}
Half-Life of the Conversion Process	$ln(2)/K_{met}$	0.525 hrs
Amount of Metabolite Formed in 5 Half-Lives	*	0.6 µmoles
Rate of Metabolite Formation	0.6/2.63	0.2 µmoles/hr

Figure S10. Conversion of valine-compound #7 to compound #7. (A) Flow chart of pharmacokinetic modeling scheme integrating single IV and PO doses of both compound #7 and valine compound #7. B1, bioavailability of valine-compound #7; K12, first order absorption rate constant of valine compound 7; Q1 and Q2, inter-compartmental clearance; CL, clearance of valine-compound #7; Fm, fraction of valine-compound #7 metabolized to compound #7; CLp, clearance of the compound #7; B2, bioavailability of compound #7; K64, first order absorption rate constant of compound 7. (B) Goodness of fit plots and model estimated parameters of the PopPK model. Individual predicted versus observed concentration (top left panel); Population predicted versus observed concentration (top right panel); Conditional weighted residuals versus population predicted concentration (bottom left panel); Conditional weighted residuals versus time (bottom right panel); Theta- Model estimated population parameters. (C) Table outline of the model estimated parameters and equations used to derive the rate of metabolite formation. * is calculated from pharmacokinetic equation for metabolite formation as shown in Figure S8.

A

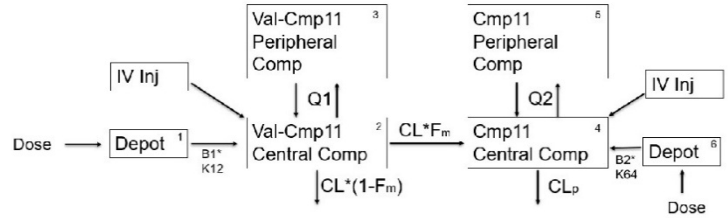

B

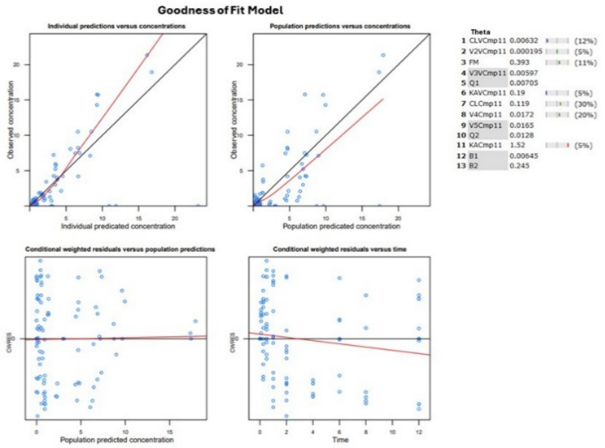

C

**Parameter**	**Source**	**Estimate**
Clearance of Valine-Compound #11 (Cl_{V11})	PopPK Model	0.006 L/hr
Volume of Central Valine-Compound #11 Compartment (V_2)	PopPK Model	0.000195 L
First-Order Rate of Elimination (K_{el})	$Cl_{VN} \div V_2$	30.11 hr^{-1}
Fraction Metabolized (F_m)	PopPk Model	0.39
First-Order Rate of Metabolite Formation (K_{met})	$F_m \times K_{el}$	11.9 hr^{-1}
Half-Life of the Conversion Process	$ln(2)/K_{met}$	0.05 hrs
Amount of Metabolite Formed in 5 Half-Lives	*	0.041 µmoles
Rate of Metabolite Formation	0.041/0.25	0.1 µmoles/hr

Figure S11. Conversion of valine-conjugated compound #11 to compound #11. (A) Flow chart of pharmacokinetic modeling scheme integrating single IV and PO doses of both compound #11 and valine-compound 11. B1, bioavailability of valine-compound #11; K12, first order absorption rate constant of valine-compound #11; Q1 and Q2, inter-compartmental clearance; CL, clearance of valine-compound #11; Fm, fraction of valine-compound #11 metabolized to compound #11; CLp, clearance of the compound #11; B2, bioavailability of compound #11; K64, first order absorption rate constant of compound #11. (B) Goodness of fit plots and model estimated parameters of the PopPK model. Individual predicted versus observed concentration (top left panel); Population predicted versus observed concentration (top right panel); Conditional weighted residuals versus population predicted concentration (bottom left panel); Conditional weighted residuals versus time (bottom right panel); Theta- Model estimated population parameters. (C) Table outline of the model estimated parameters and equations used to derive the rate of metabolite formation. * is calculated from pharmacokinetic equation for metabolite formation as shown in Figure S8.
