# Supplementary material for: Valine–Niclosamide for Treatment of Androgen Receptor Splice Variant-Positive Hepatocellular Carcinoma
Source: Cancers (Basel). 2025 Jul 31;17(15):2535. doi: 10.3390/cancers17152535 (PMC12346198; doi:10.3390/cancers17152535)

Scheme S1:

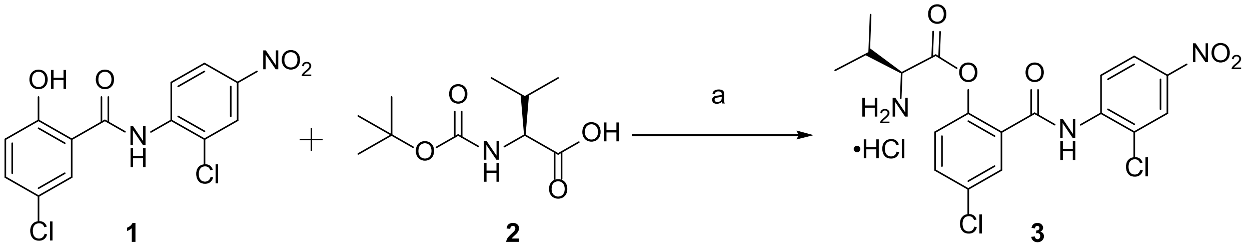

Valine-Niclosamide (**JFR-I-51-01**)

Scheme S1. Synthesis of valine-niclosamide (**3**). Reagents and conditions: a (i) **1**(1.0 eq), **2** (1.5 eq), EDCI (1.5 eq), DMAP (0. 1 eq), THF, overnight, rt; ii) 4N HCl in dioxane, overnight, rt.

Experimental procedure.

1). Synthesis of 4-chloro-2-((2-chloro-4-nitrophenyl)carbamoyl)phenyl L-valinate ·Hydrochloride (**3, Valine-Niclosamide, JFR-I-51-01**):

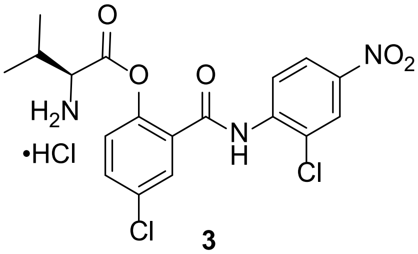

3

To the solution of 5-*chloro-N-(2-chloro-4-nitrophenyl)-2-hydroxybenzamide* in THF, was added EDCI (1.5 eq), DMAP (0. 1 eq), and stirred for 15 min. Then, Boc-L-valine (1.5 eq) was added to the reaction mixture and stirred for overnight. The reaction mixture was evaporated and diluted with EtOAc and water. The compound extracted with EtOAC (3×50 mL) and dried on Na_2SO_4 and concentrated on reduced vapor pressure. The compound was purified by combi flash using 0-15% EtOAc in Hexanes gradient. The pure fractions concentrated and converted as a HCl salt using 4 equivalent of HCl in dioxane (42% yield, Colorless solid): ^{1}HNMR (400 MHz, DMSO-d_6) δ 11.98 (s, 1H), 10.18 (s, 1H), 8.97 (d, J = 8.0 Hz, 1H), 8.37 (d, J = 2.5 Hz, 1H), 8.24 - 8.21 (m, 2H), 7.99 (d, J = 2.6 Hz, 1H), 7.45 (dd, J = 8.7, 2.6 Hz, 1H), 6.99 (d, J = 8.7 Hz, 1H), 4.85 (t, J = 6.9 Hz, 1H), 2.33 - 2.23 (m, 1H), 1.04 - 0.89 (m, 6H); LC-MS (ESI), $C_{18}H_{17}Cl_2N_3O_5$: [M+H] 426.033.

Recharge Acct #GR123994#

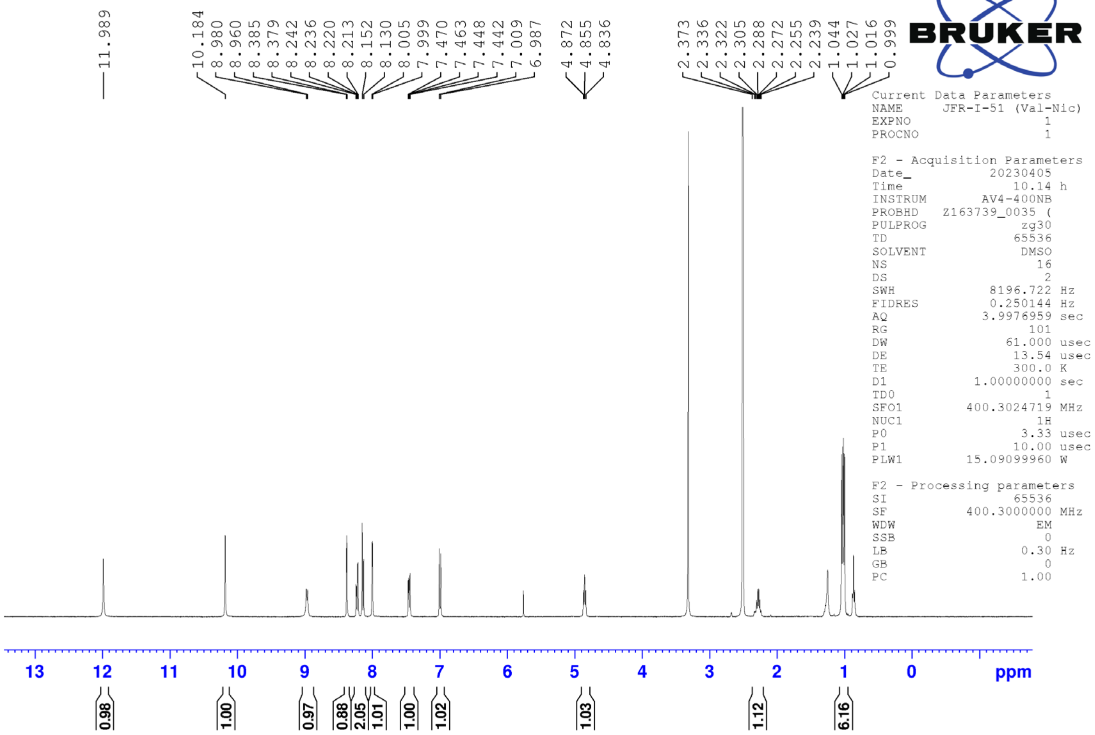

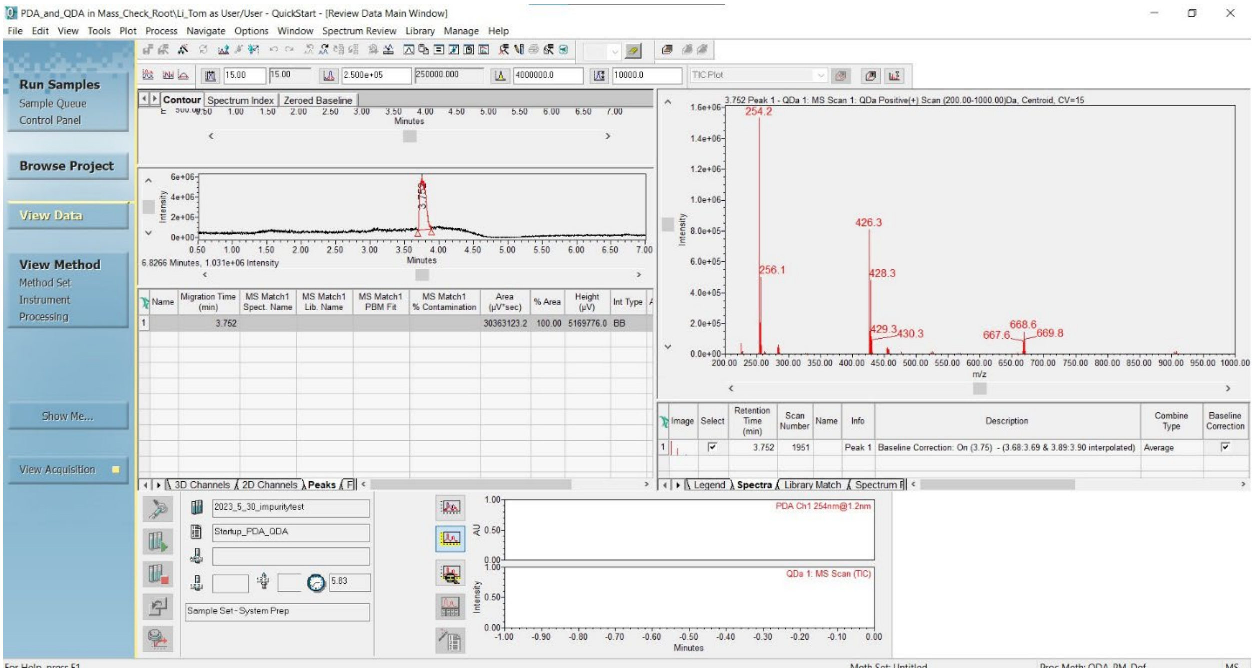

Scheme S2:

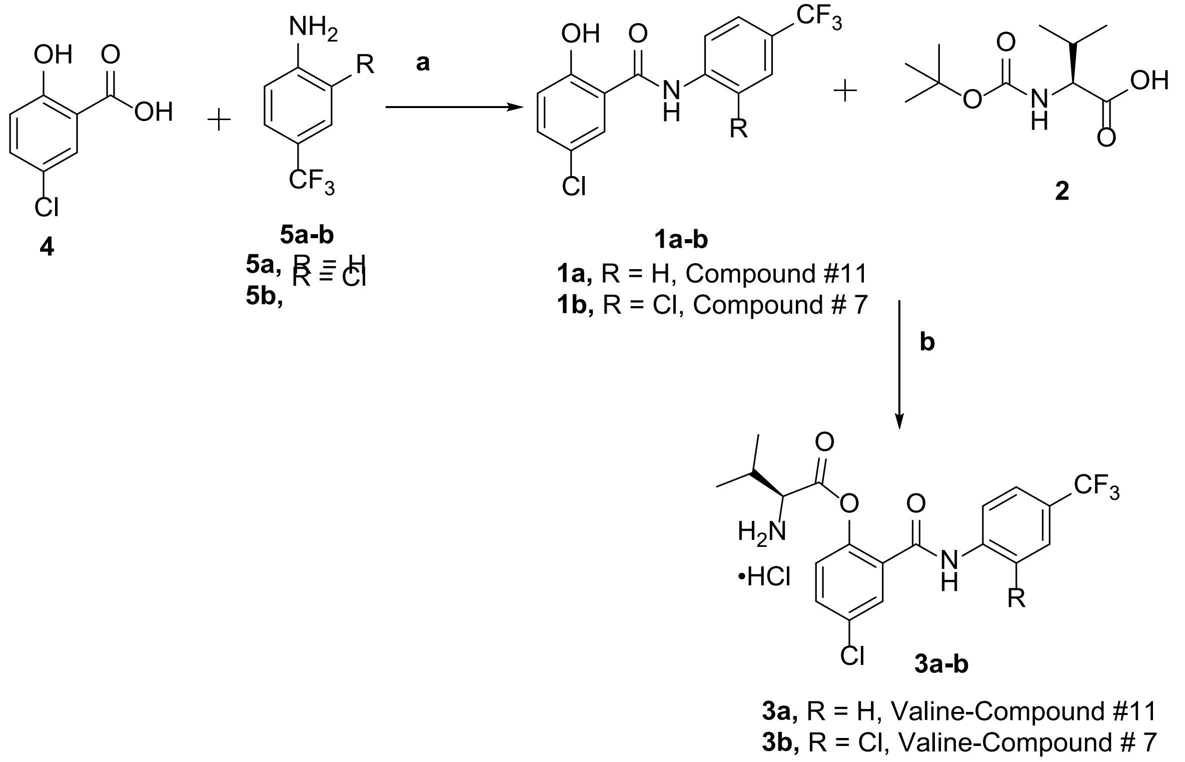

Scheme S2. Synthesis of valine-compounds #**7** & #11. Reagents and conditions: (a) **4** (1.0 eq), PCl_3, (0.4 eq), 120° C, *m*-xylene, 3 hours; **b** (i) **1** (1.0 eq), **2** (1.5 eq), EDCI (1.5 eq), DMAP (0. 1 eq), THF, overnight, rt; ii) 4N HCl in dioxane, overnight, rt.

Procedure a: To the solution of corresponding carboxylic acids (1.0 eq) in *m*-xylene was added corresponding anilines (1.1 eq) and heated to 110°C. After 20 min, PCl_3 (0.4 eq) was added to reaction mixture and raised the temperature to 120 °C and stirred for another 3 hours. The reaction mixture was brought to 80°C and diluted with water. Major of the compounds are precipitated and filtered off, washed with Hexane and pet-ether and dried. The compounds which are not precipitated, followed the Combi flash purification by using EtOAc in Hexane system.

Procedure b: To the solution of corresponding salicylanilides in THF, was added EDCI (1.5 eq), DMAP (0. 1 eq), and stirred for 15 min. Then, Boc-L-valine (1.5 eq) was added to the reaction mixture and stirred for overnight. The reaction mixture was evaporated and diluted with EtOAc and water. The compound extracted with EtOAC (3×50 mL) and dried on Na_2SO_4 and concentrated on reduced vapor pressure. The compound was purified by combi flash using 0-15% EtOAc in Hexanes gradient. The pure fractions concentrated and converted as a HCl salt using 4 equivalents of HCl in dioxane

2). 5-chloro-2-hydroxy-N-(4-(trifluoromethyl)phenyl)benzamide (**1a**, **Compound #11, SOH-I-158**)

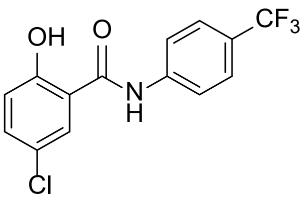

Compound #11

(White solid, 58% yield): ^{1}HNMR (400 MHz, DMSO-d_6) δ 11.5 (s, 1H), 10.6 (s, 1H), 7.94 (d, J = 8.4 Hz, 2H), 7.88 (d, J = 2.6 Hz, 1H), 7.74 (d, J = 8.6 Hz, 2H), 7.47 (dd, J = 8.8 Hz, 2.7 Hz, 1H), 7.03 (d, J = 8.7 Hz, 1H); LC-MS (ESI); [M-H]: 313.9

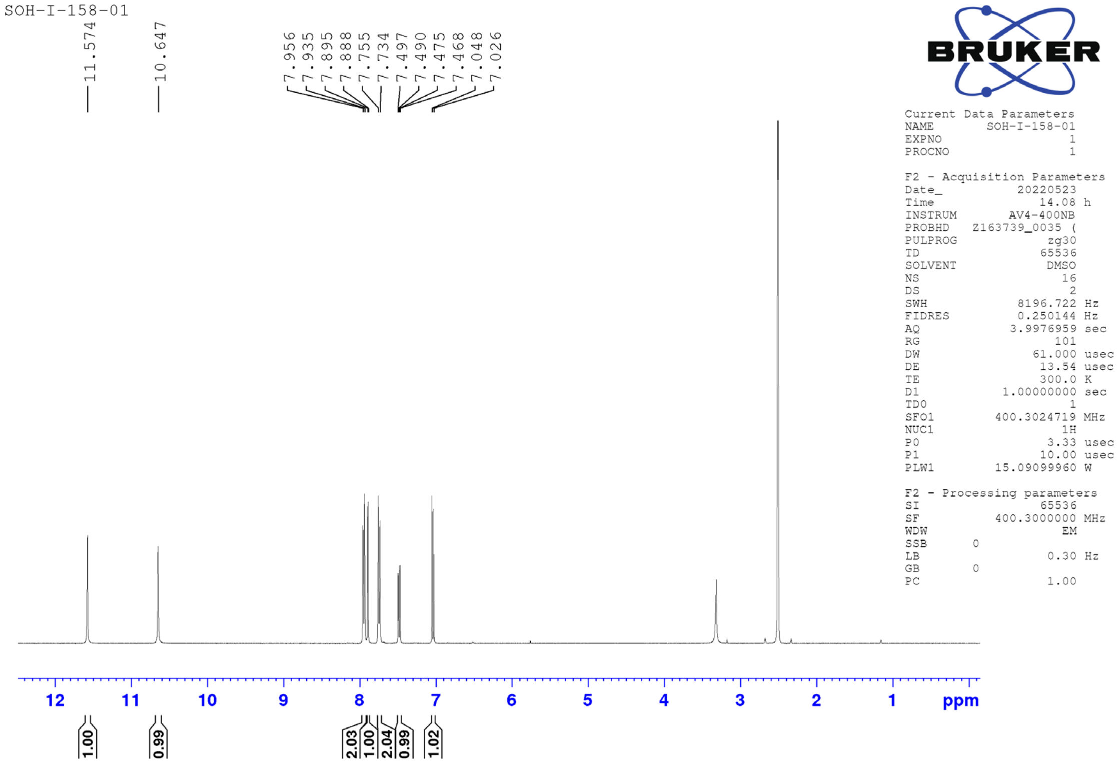

Vial:1:14	ID:mohammed210_149-4-20240919-1212	File:mohammed210_149-4-20240919-1212_COD
Date:19-Sep-2024	Time:12:37:07	Description:Nic-11
Method:C:\MassLynx\OpenLynx_Methods\ESI-_100-1250+PDA.olp		Instrument:ACQ-QDA#KBD6021
MS Method:OA_ESI-_Default	Inlet Method:OA_Default	Detectors:Waters Acquity PDA

Printed: Thu Sep 19 13:03:11 2024

Sample Report (continued):

Sample 4 Nic-11 19-Sep-2024 12:37:07 File: mohammed210_149-4-20240919-1212_COD

1: MS ES- :TIC Smooth (SG, 2x3) 4.1e+006

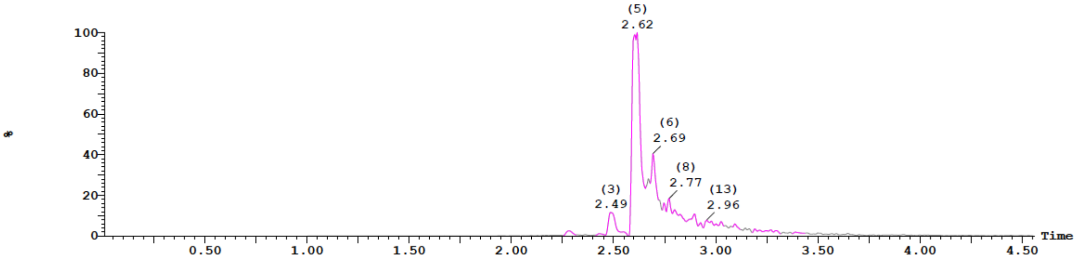

2: UV Detector: TAC: Wavelength Range: (210 - 499) 1.825e+1
Range: 1.825e+1

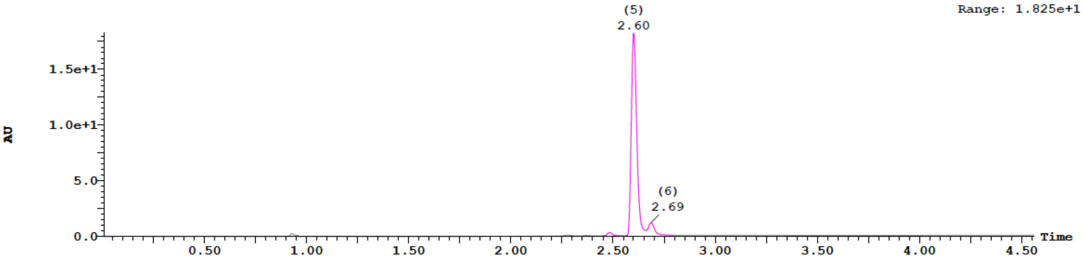

Sample Report (continued):

Peak ID 5 **Time** 2.60
5:(Time: 2.60) Combine (778) 2:UV Detector 3.144e-1 AU

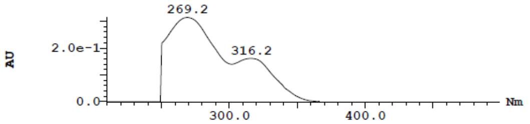

Peak ID 6 **Time** 2.69
6:(Time: 2.69) Combine (476:536-(446:475+537:566)) 1:MS ES- 7.6e+005

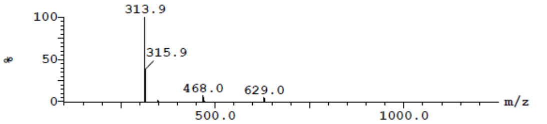

Peak ID 6 **Time** 2.69
6:(Time: 2.69) Combine (804) 2:UV Detector 4.947e-2 AU

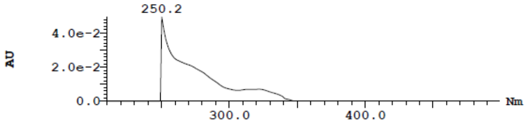

Peak ID 7 **Time** 2.75
7:(Time: 2.75) Combine (494:554-(464:493+555:584)) 1:MS ES- 5.4e+004

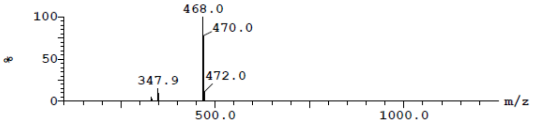

Peak ID 8 **Time** 2.77
8:(Time: 2.77) Combine (502:562-(472:501+563:592)) 1:MS ES- 5.4e+004

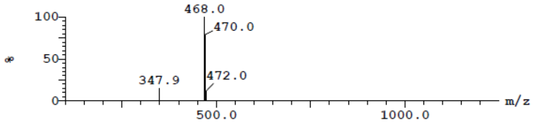

Peak ID 9 **Time** 2.80
9:(Time: 2.80) Combine (510:570-(480:509+571:600)) 1:MS ES- 7.8e+003

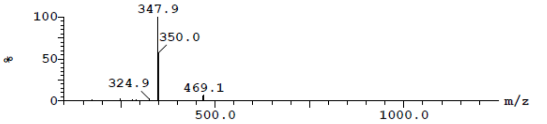

3). Synthesis of 4-chloro-2-((4-(trifluoromethyl)phenyl)carbamoyl)phenyl L-valinate ·Hydrochloride (**3a, Valine-Compound #11, JFR-I-58-01**)[1]

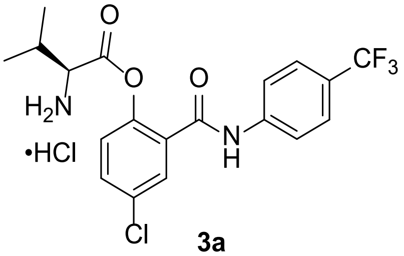

3a

Colorless solid, 55% yield,: ^{1}HNMR (400 MHz, DMSO-d6) δ 10.96 (s, 1H), 8.73 (brs, 2H), 7.93 - 7.87 (m, 3H), 7.76 - 7.72 (m, 3H), 7.47 (d, *J* = 9.9 Hz, 1H), 4.12 (brs, 1H), 3.51 - 3.50 (brs, 1H), 2.33 - 2.26 (m, 1H), 0.99 - 0.96 (m, 6H); ^{13}CNMR (100 MHz, DMSO-d_6) δ 167.8, 163.4, 146.0, 142.8, 132.0, 131.6, 131.2, 129.2, 126.5, 126.4, 125.6, 120.1, 57.9, 29.6, 18.2; LC-MS (ESI), $C_{19}H_{18}ClF_3N_2O_3$, [M+ H]: 415.3

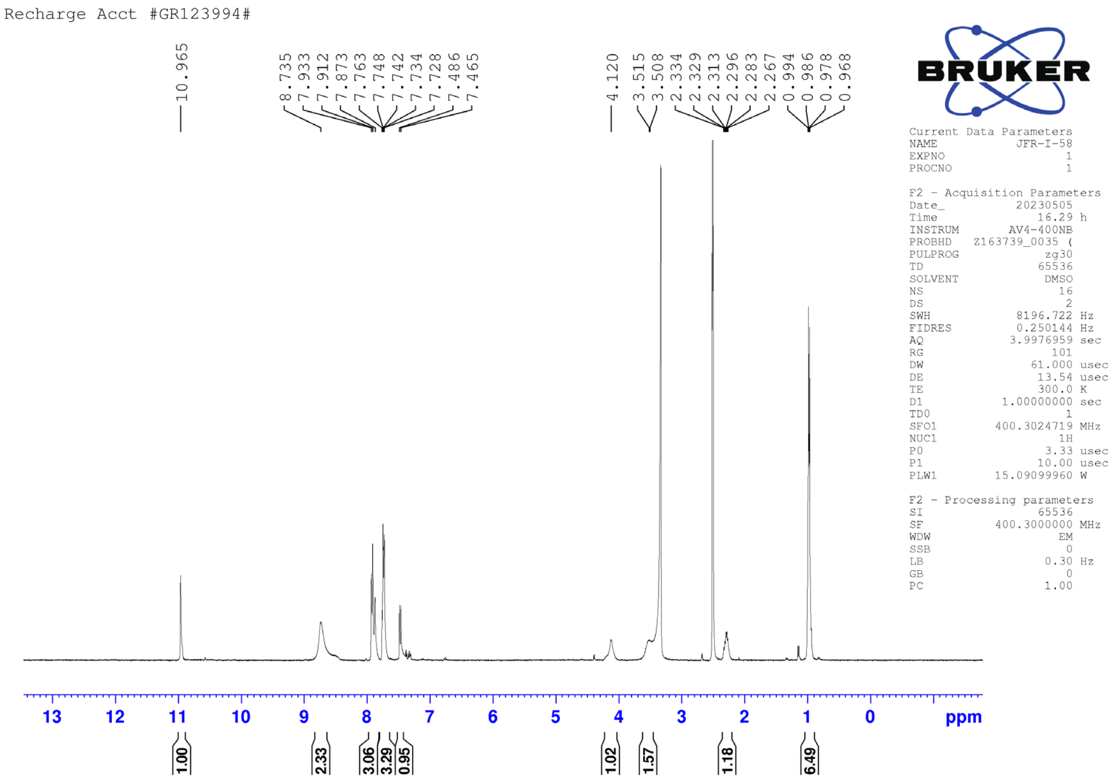

[1] https://doi.org/10.1016/j.bmc.2013.08.029

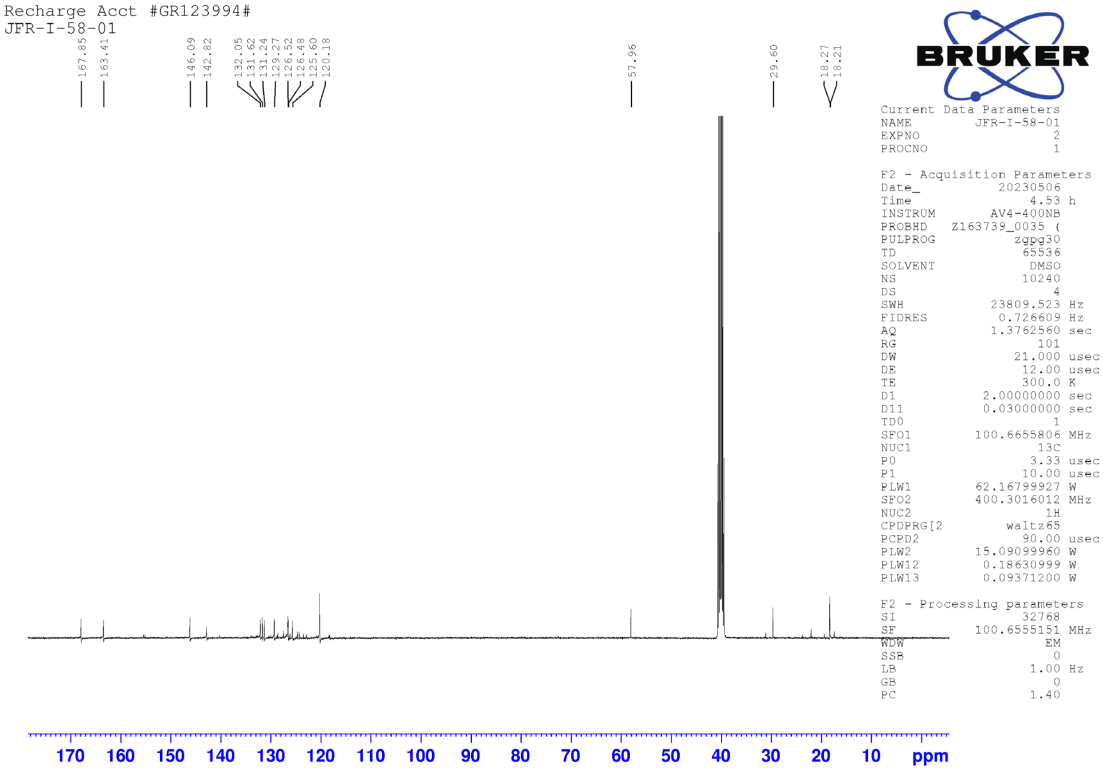

Recharge Acct #GR123994#
JFR-I-58-01
167.85
163.41
146.09
142.82
132.05
131.62
131.24
129.27
126.52
126.48
125.60
120.18
57.96
29.60
18.27
18.21
BRUKER
Current Data Parameters
NAME JFR-I-58-01
EXPNO 2
PROCNO 1
F2 - Acquisition Parameters
Date_ 20230506
Time 4.53 h
INSTRUM AV4-400NB
PROBHD Z163739_0035 (
PULPROG zgpg30
TD 65536
SOLVENT DMSO
NS 10240
DS 4
SWH 23809.523 Hz
FIDRES 0.726609 Hz
AQ 1.3762560 sec
RG 101
DW 21.000 usec
DE 12.00 usec
TE 300.0 K
D1 2.00000000 sec
D11 0.03000000 sec
TD0 1
SFO1 100.6655806 MHz
NUC1 13C
P0 3.33 usec
P1 10.00 usec
PLW1 62.16799927 W
SFO2 400.3016012 MHz
NUC2 1H
CPDPRG[2 waltz65
PCPD2 90.00 usec
PLW2 15.09099960 W
PLW12 0.18630999 W
PLW13 0.09371200 W
F2 - Processing parameters
SI 32768
SF 100.6555151 MHz
WDW EM
SSB 0
LB 1.00 Hz
GB 0
PC 1.40
170 160 150 140 130 120 110 100 90 80 70 60 50 40 30 20 10 ppm

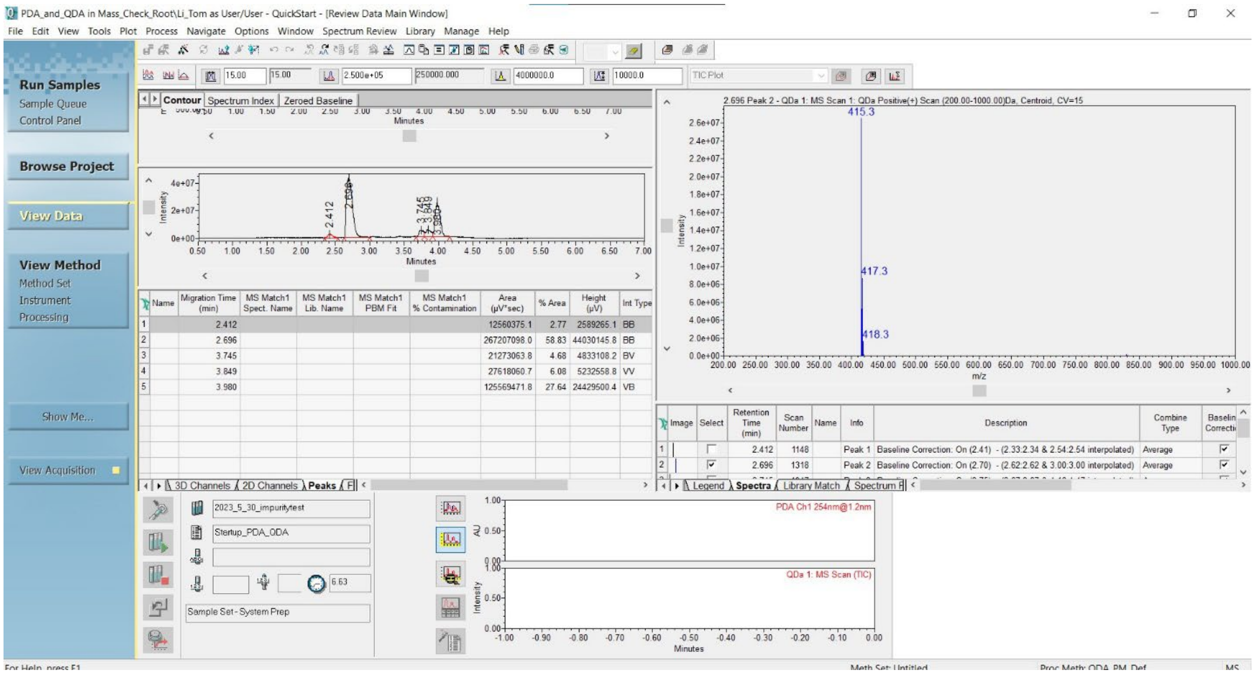

PDA_and_QDA in Mass_Check_Root\Li_Tom as User/User - QuickStart - [Review Data Main Window]
Run Samples
Browse Project
View Data
View Method
2.696 Peak 2 - QDa 1: MS Scan 1: QDa Positive(+) Scan (200.00-1000.00)Da, Centroid, CV=15
415.3
417.3
418.3
m/z
Minutes
Name | Migration Time (min) | Area (µV*sec) | % Area | Height (µV) | Int Type
1 | 2.412 | 12560375.1 | 2.77 | 2589265.1 | BB
2 | 2.696 | 267207098.0 | 58.83 | 44030145.8 | BB
3 | 3.745 | 21273063.8 | 4.68 | 4833108.2 | BV
4 | 3.849 | 27618060.7 | 6.08 | 5232558.8 | VV
5 | 3.980 | 125569471.8 | 27.64 | 24429500.4 | VB
2023_5_30_impuritytest
Startup_PDA_QDA
Sample Set - System Prep
PDA Ch1 254nm@1.2nm
QDa 1: MS Scan (TIC)

4). *5-chloro-N-(2-chloro-4-(trifluoromethyl)phenyl)-2-hydroxybenzamide* (**1b, Compound #7, SOH-I-167-01**)

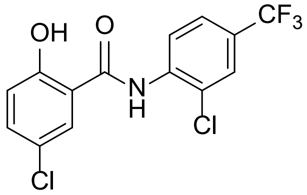

Compound # 7

(White solid, 51% yield): ^{1}HNMR (400 MHz, DMSO-d6) δ 12.4 (s, 1H), 11.17 (s, 1H), 8.73 (d, *J* = 8.4 Hz, 1H), 8.0 - 7.98 (m, 2H), 7.79 (dd, *J* = 8.7 Hz, 1.6 Hz, 1H), 7.53 (dd, *J* = 8.7 Hz, 2.8 Hz, 1H), 7.10 (d, *J* = 4.3 Hz, 1H); LC-MS (ESI); [M-H]: 348.0

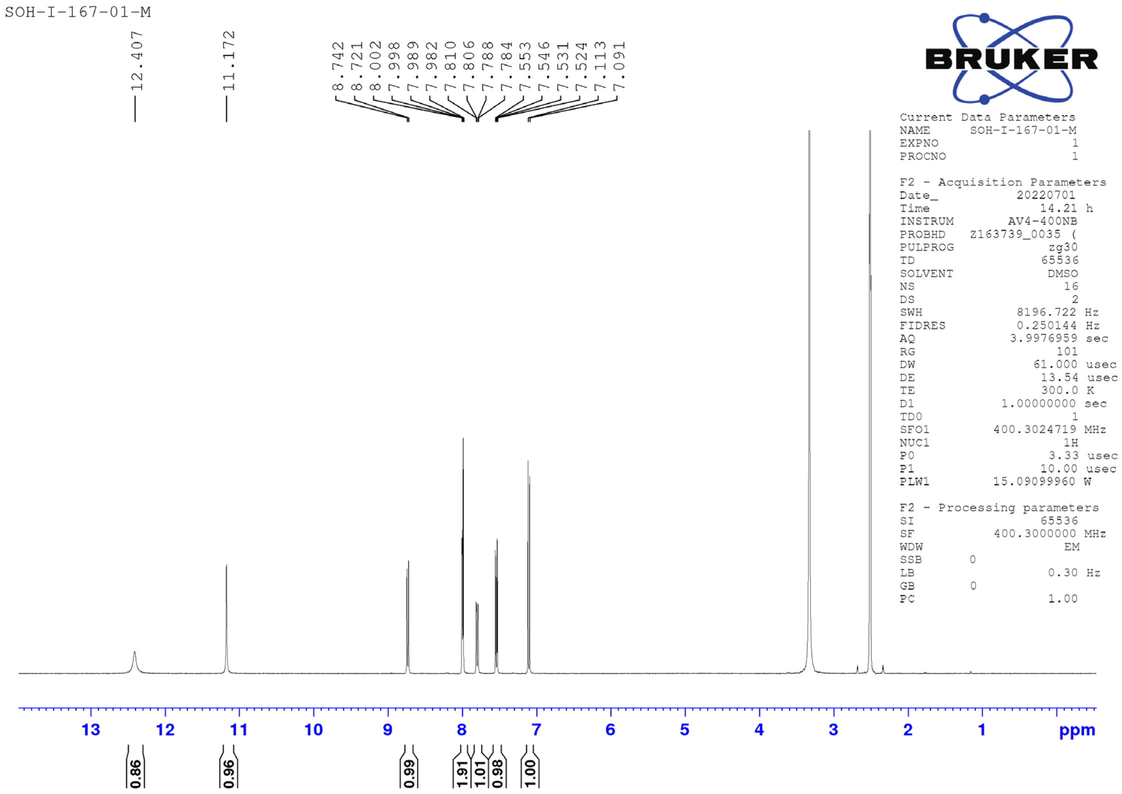

Vial:2:2
Date:16-Sep-2024
Method:C:\MassLynx\OpenLynx_Methods\ESI-_100-1250+PDA.olp
MS Method:OA_ESI-_Default

ID:mohammed210_147-07-20240916-1113
Time:12:47:55
Inlet Method:OA_Default

File:mohammed210_147-07-20240916-1113_COD
Description:SOH-I-167-01
Instrument:ACQ-QDA#KBD6021
Detectors:Waters Acquity PDA

Printed: Mon Sep 16 13:31:21 2024

Sample Report (continued):

Sample 7 SOH-I-167-01 16-Sep-2024 12:47:55 File: mohammed210_147-07-20240916-1113_COD

1: MS ES- :TIC Smooth (SG, 2x3) 3.9e+006

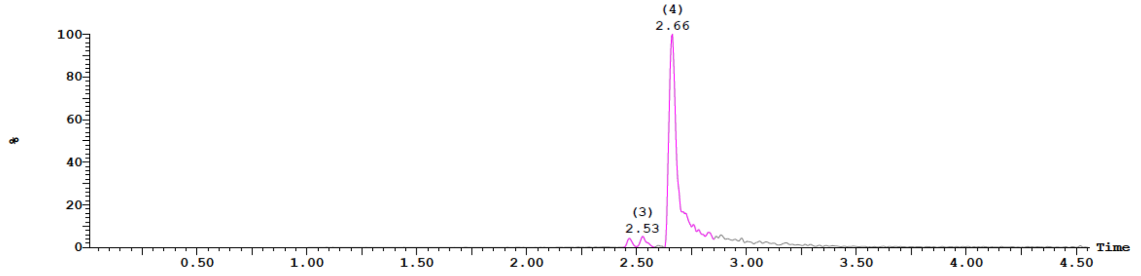

2: UV Detector: TAC: Wavelength Range: (210 - 499) 9.956
Range: 9.956

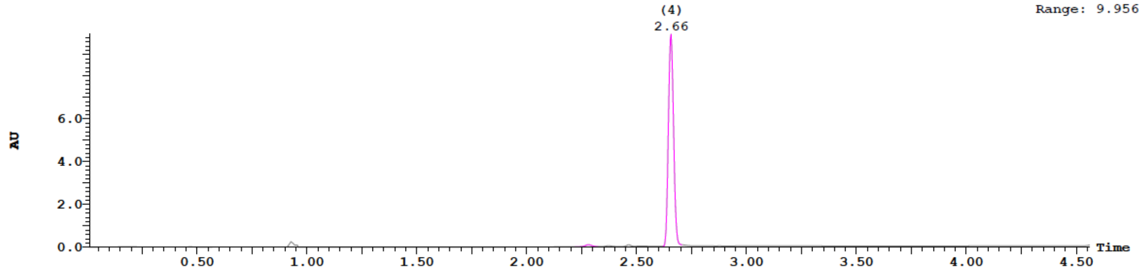

Sample Report (continued):

Peak ID 1 **Time** 2.28
1: (Time: 2.28) Combine (354:414-(324:353+415:444)) 1:MS ES- 7.2e+002

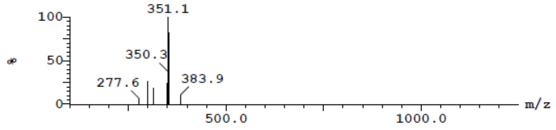

Peak ID 1 **Time** 2.28
1: (Time: 2.28) Combine (682) 2:UV Detector 1.195e-2 AU

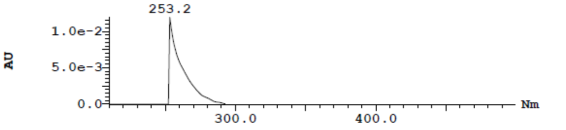

Peak ID 2 **Time** 2.47
2: (Time: 2.47) Combine (410:470-(380:409+471:500)) 1:MS ES- 1.6e+004

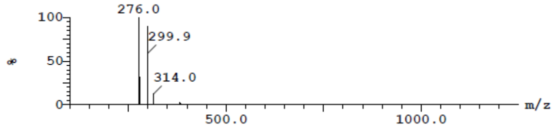

Peak ID 3 **Time** 2.53
3: (Time: 2.53) Combine (428:488-(398:427+489:518)) 1:MS ES- 1.7e+004

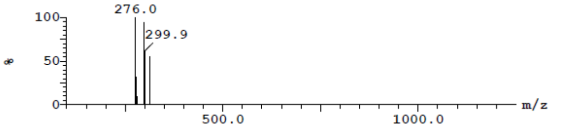

Peak ID 4 **Time** 2.66
4: (Time: 2.66) Combine (467:527-(437:466+528:557)) 1:MS ES- 3.8e+005

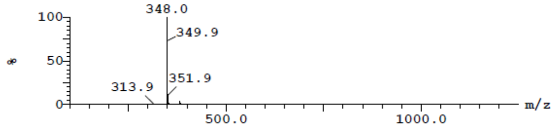

Peak ID 4 **Time** 2.66
4: (Time: 2.66) Combine (795) 2:UV Detector 1.924e-1 AU

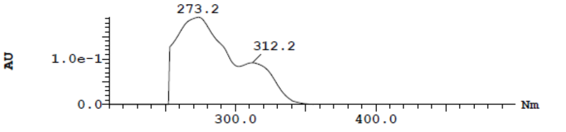

5). Synthesis of 4-chloro-2-((2-chloro-4-(trifluoromethyl)phenyl)carbamoyl)phenyl L-valinate ·Hydrochloride (**3b, Valine-Compound #7, SOH-III-106-02**):

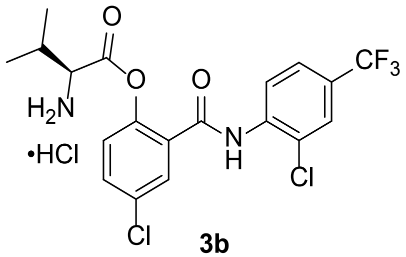

3b

Colorless solid, 43% yield,: ^{1}HNMR (400 MHz, CD_3OD) δ 8.08 (b, *J* = 8.4 Hz, 1H), 7.90 - 7.74 (m, 2H), 7.69 - 7.66 (m, 2H), 7.35 (d, *J* = 8.7 Hz, 1H), 4.22 (d, *J* = 4.2 Hz, 1H), 2.55- 2.47 (m, 1H), 1.17 - 1.11 (m, 6H); ^{13}CNMR (100 MHz, CD_3OD) δ 168.7, 165.4, 147.6, 139.1, 133.5, 130.3, 129.4, 127.9, 127.9, 127.7, 126.0, 125.5, 120.5, 59.5, 30.7, 18.6, 17.9; LC-MS (ESI), $C_{19}H_{17}Cl_2F_3N_2O_3$ [M+H]: 449.2

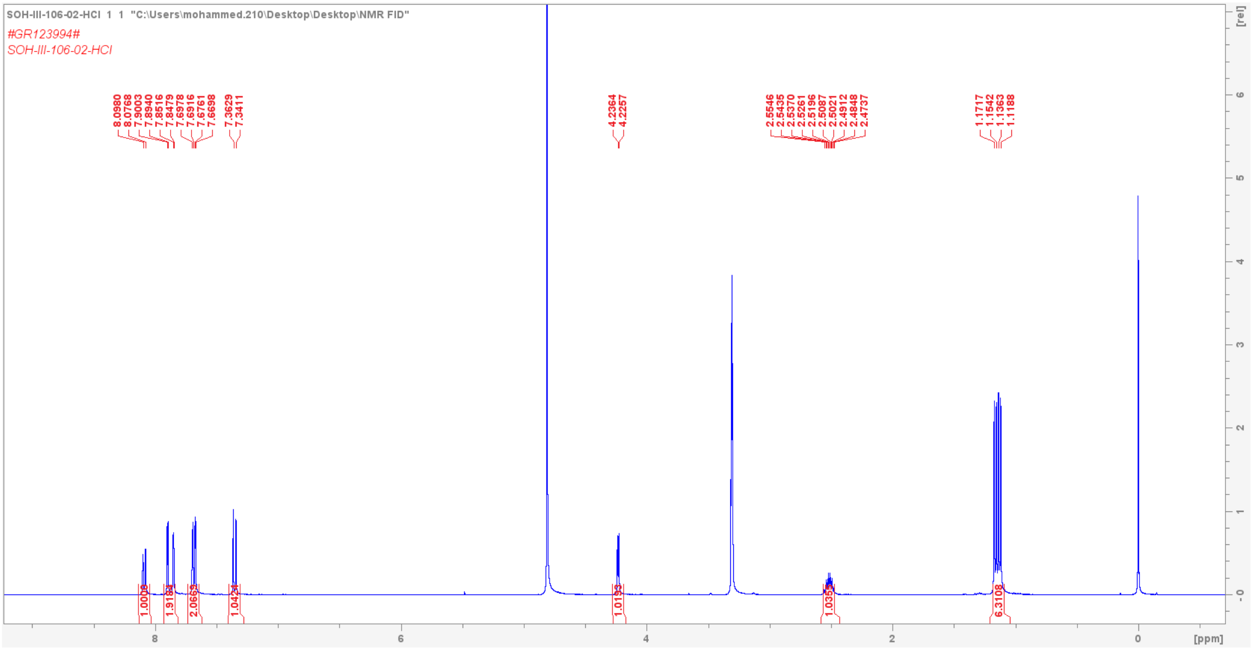

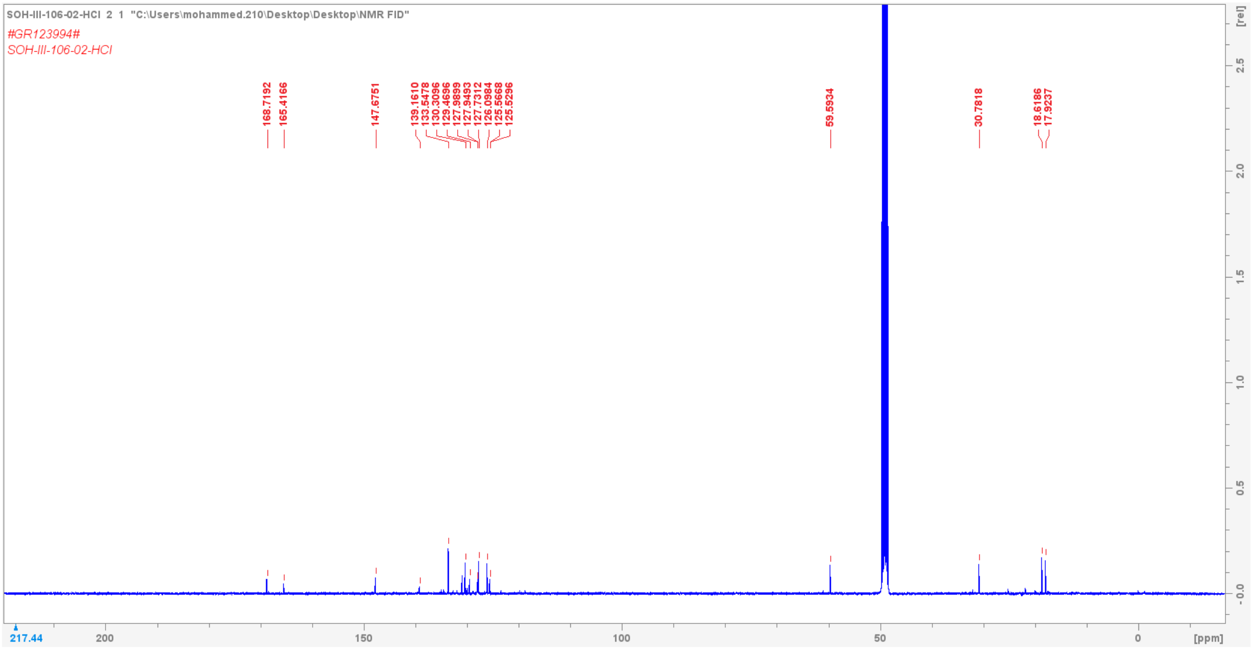

OSU College of Pharmacy Shared Instrumentation Facility

Openlynx Report – mohammed210

Vial:2:2
Date:30-Jul-2024
Method:C:\MassLynx\OpenLynx_Methods\ESI+_100-1250+PDA.olp
MS Method:OA_ESI+_Default

ID:mohammed210_134-2-20240730-1617
Time:16:24:13
Inlet Method:OA_Default

File:mohammed210_134-2-20240730-1617_COD
Description:SOH-III-106-02-HCl
Instrument:ACQ-QDA#KBD6021
Detectors:Waters Acquity PDA

Printed: Tue Jul 30 16:31:25 2024

Sample Report (continued):

Sample 2 SOH-III-106-02-HCl 30-Jul-2024 16:24:13 File: mohammed210_134-2-20240730-1617_COD

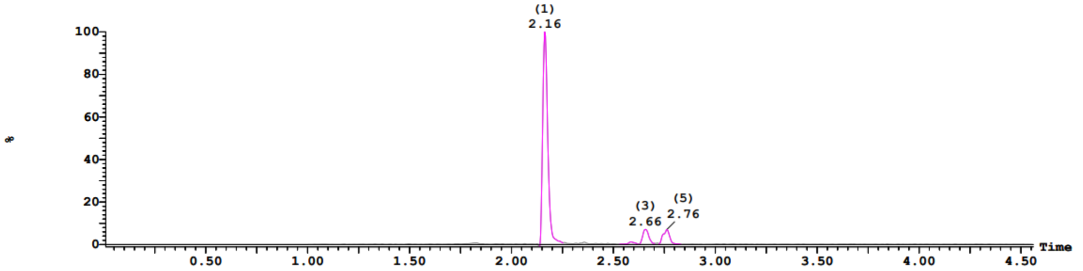

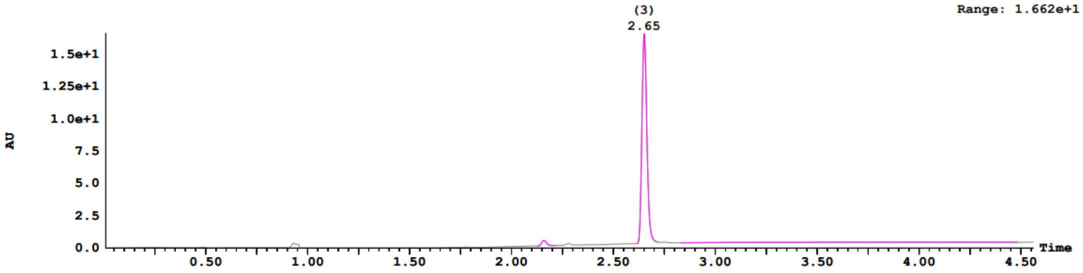

Sample Report (continued):

Peak ID **Time**
1 2.16
1: (Time: 2.16) Combine (319:379-(289:318+380:409)) 1:MS ES+
3.2e+005

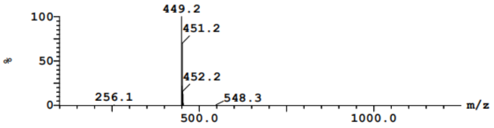

Peak ID **Time**
1 2.16
1: (Time: 2.16) Combine (646) 2:UV Detector
4.202e-2 AU

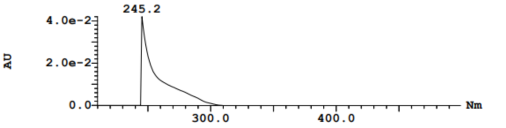

Peak ID **Time**
2 2.59
2: (Time: 2.59) Combine (446:506-(416:445+507:536)) 1:MS ES+
1.6e+004

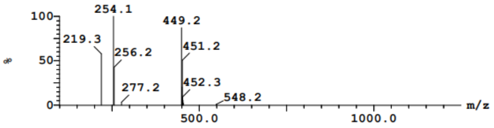

Peak ID **Time**
3 2.66
3: (Time: 2.65) Combine (465:525-(435:464+526:555)) 1:MS ES+
1.9e+004

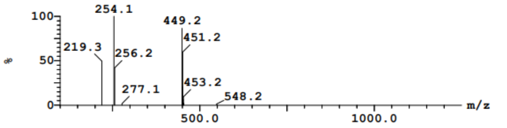

Peak ID **Time**
3 2.66
3: (Time: 2.65) Combine (793) 2:UV Detector
6.232e-1 AU

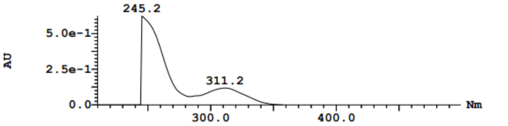

Peak ID **Time**
4 2.75
4: (Time: 2.75) Combine (494:554-(464:493+555:584)) 1:MS ES+
2.9e+004

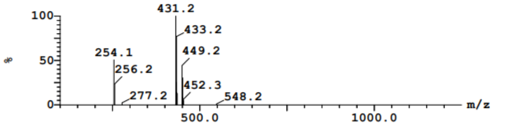

Supplement: Supplementary file 1 [file cancers-17-02535-s001.zip › Valine-Niclosamide_Manuscript_Supplemental_Schemes.pdf]
