# Supplementary figures and images for: Valine–Niclosamide for Treatment of Androgen Receptor Splice Variant-Positive Hepatocellular Carcinoma
Source: Cancers (Basel). 2025 Jul 31;17(15):2535. doi: 10.3390/cancers17152535 (PMC12346198; doi:10.3390/cancers17152535)

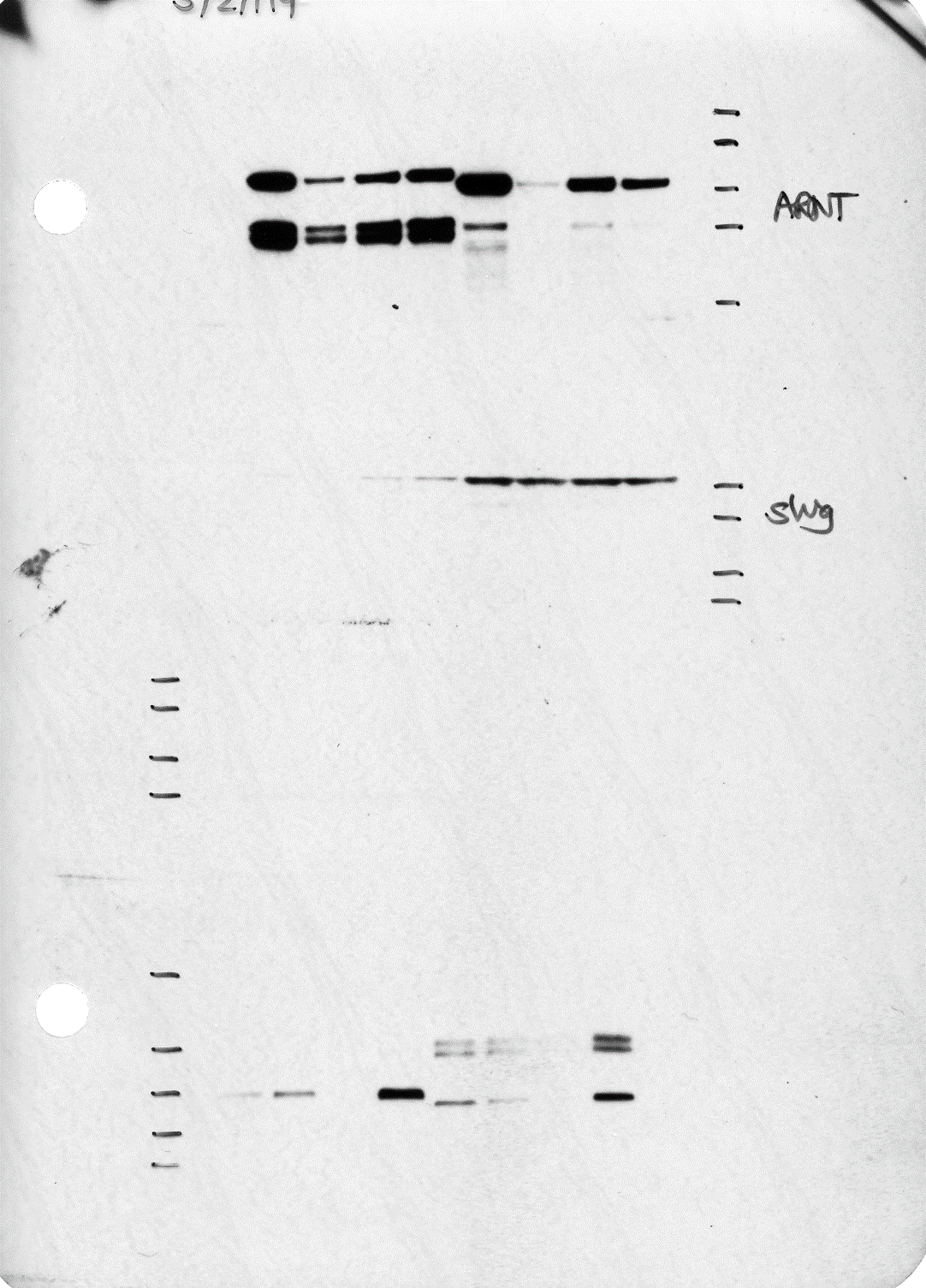

Supplement: Supplementary file 1 [file cancers-17-02535-s001.zip › Figure1B_HCCLM3_AR-NT.tif]

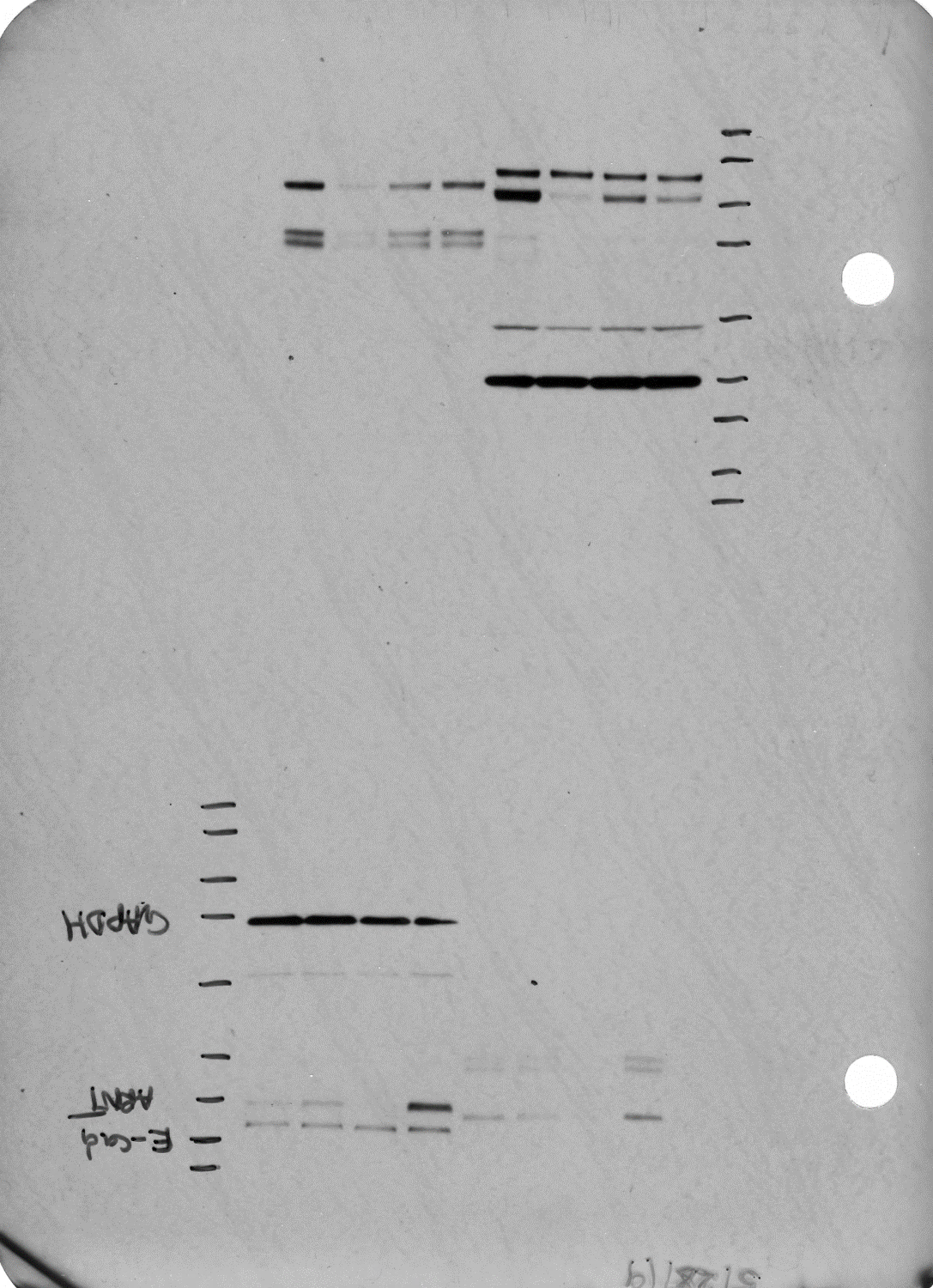

Supplement: Supplementary file 1 [file cancers-17-02535-s001.zip › Figure1B_HCCLM3_GAPDH.tif]

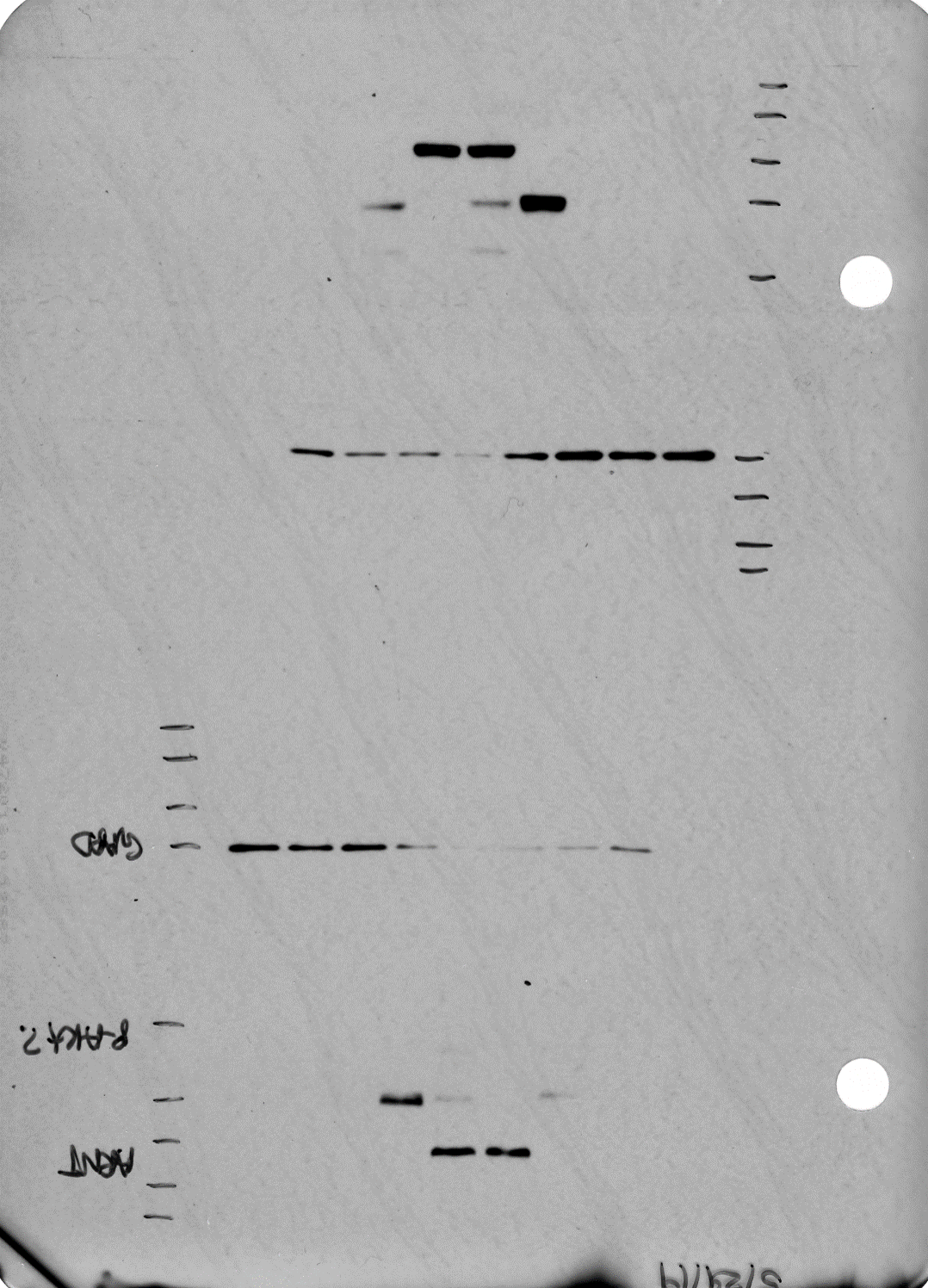

Supplement: Supplementary file 1 [file cancers-17-02535-s001.zip › Figure1B_SNU475_AR-NT_GAPDH.tif]

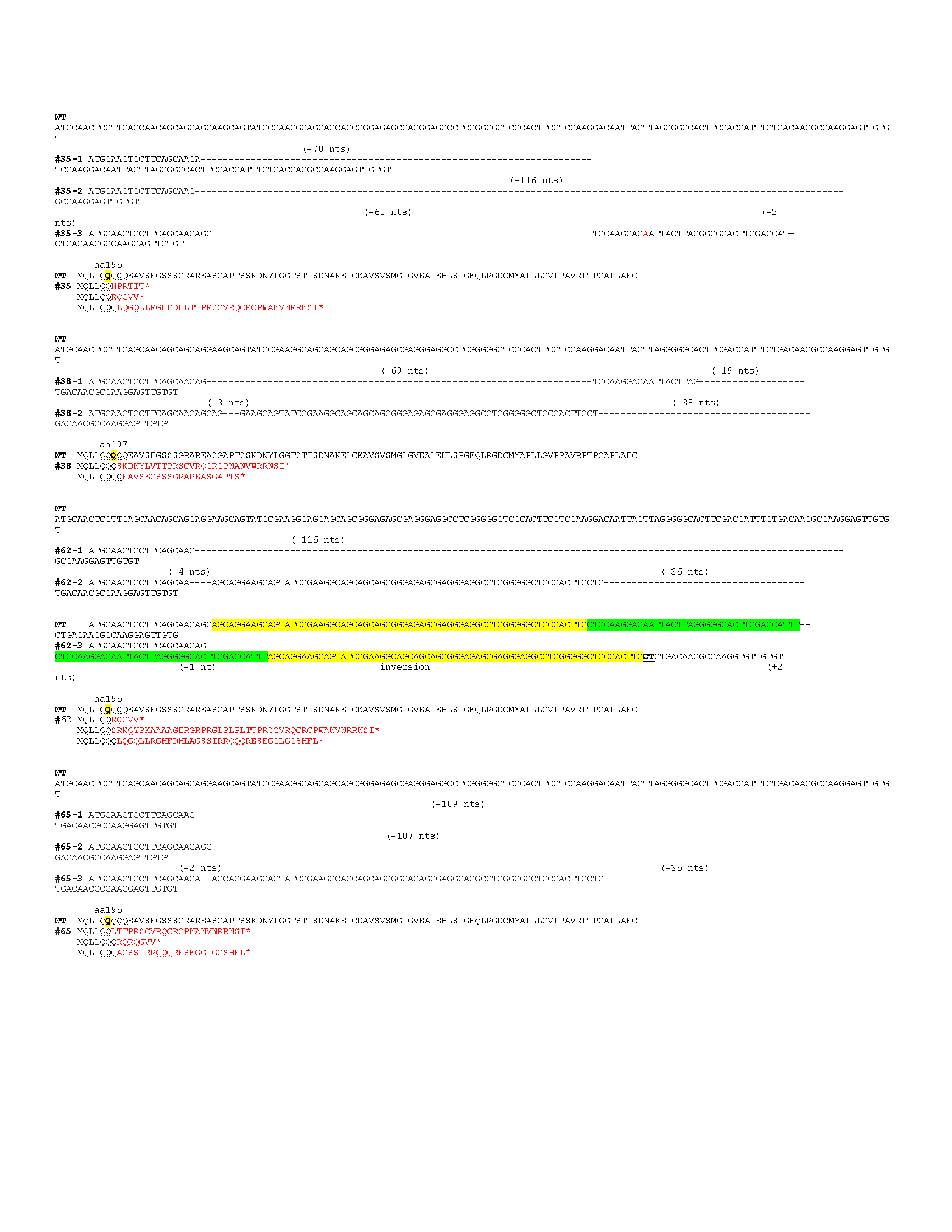

Supplement: Supplementary file 1 [file cancers-17-02535-s001.zip › Figure_S1.tif]

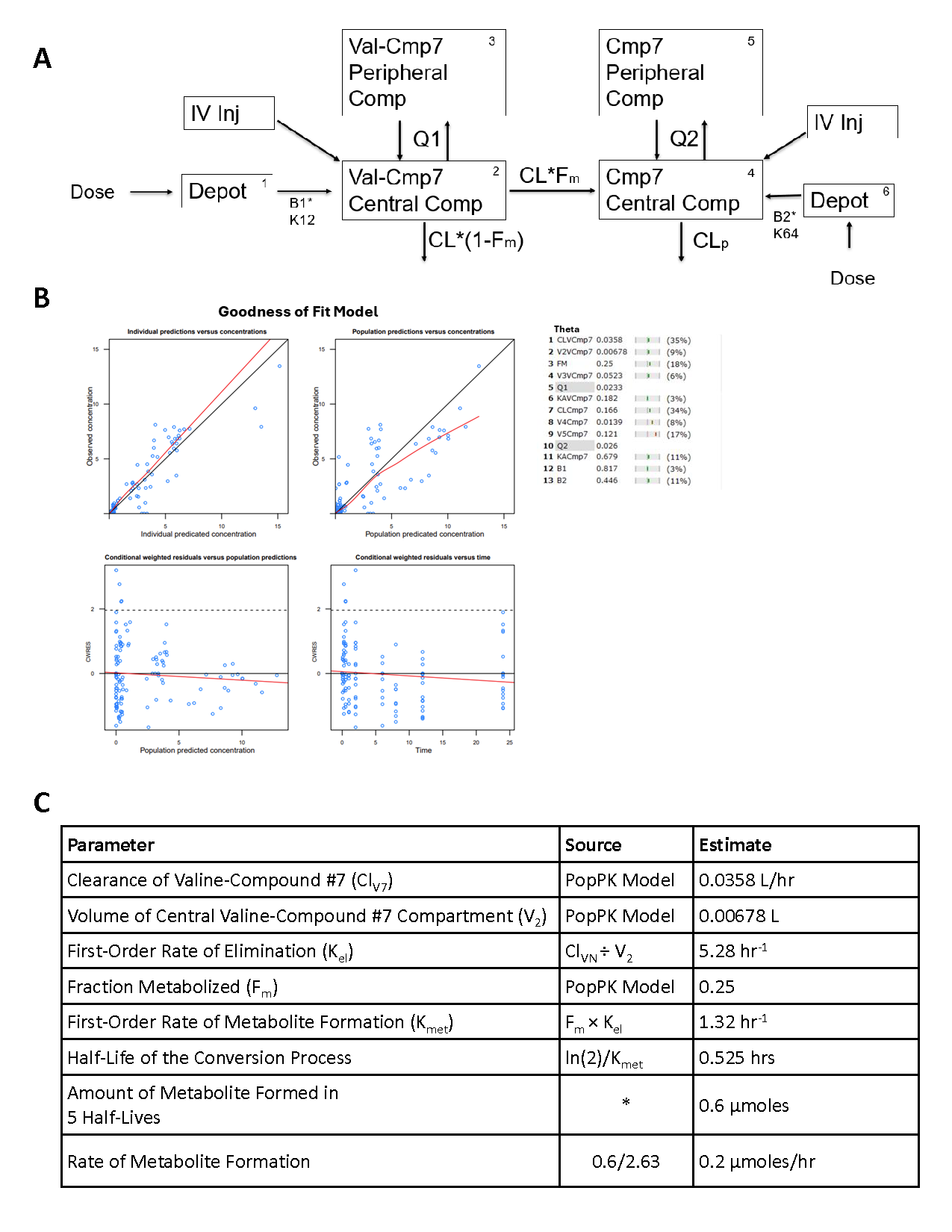

Supplement: Supplementary file 1 [file cancers-17-02535-s001.zip › Figure_S10.tif]

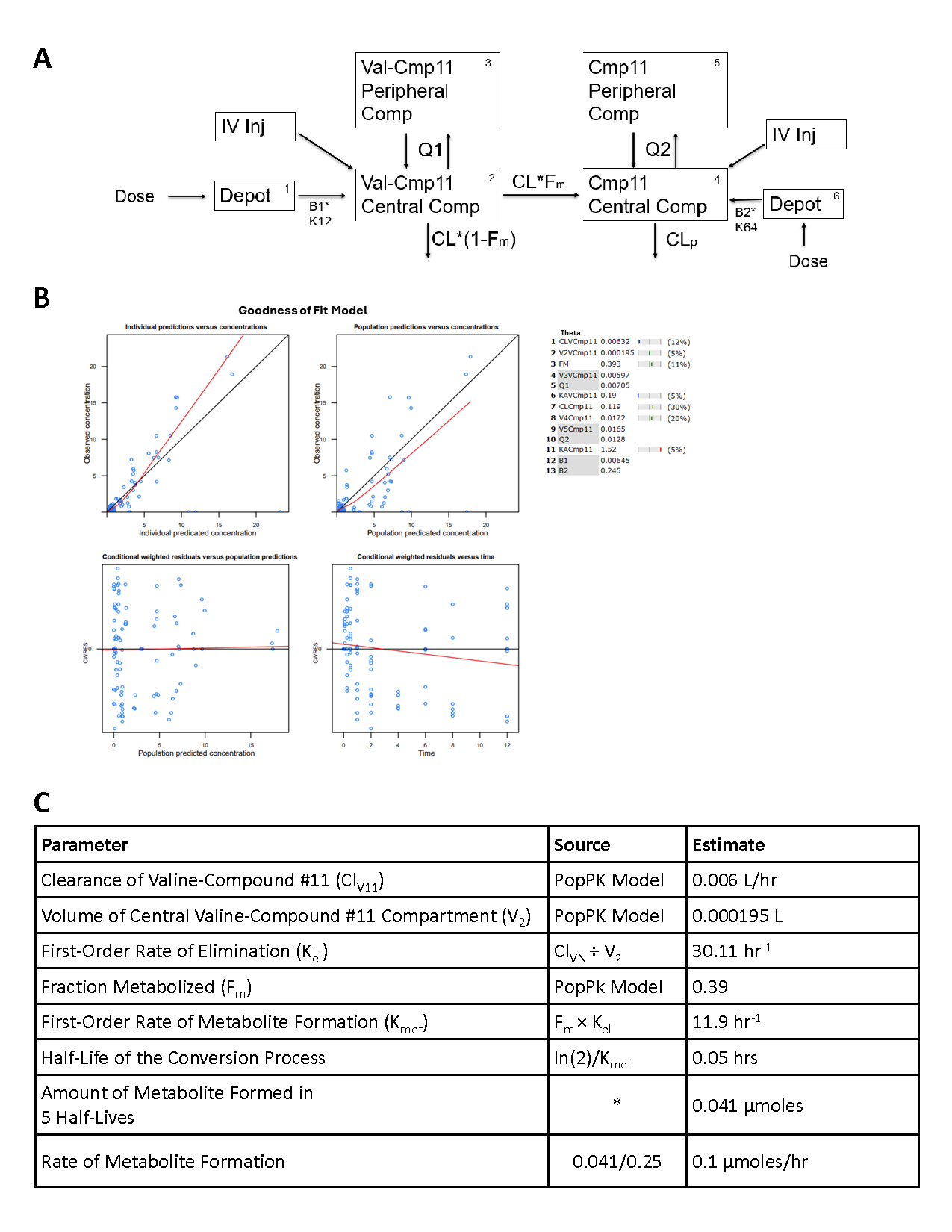

Supplement: Supplementary file 1 [file cancers-17-02535-s001.zip › Figure_S11.tif]

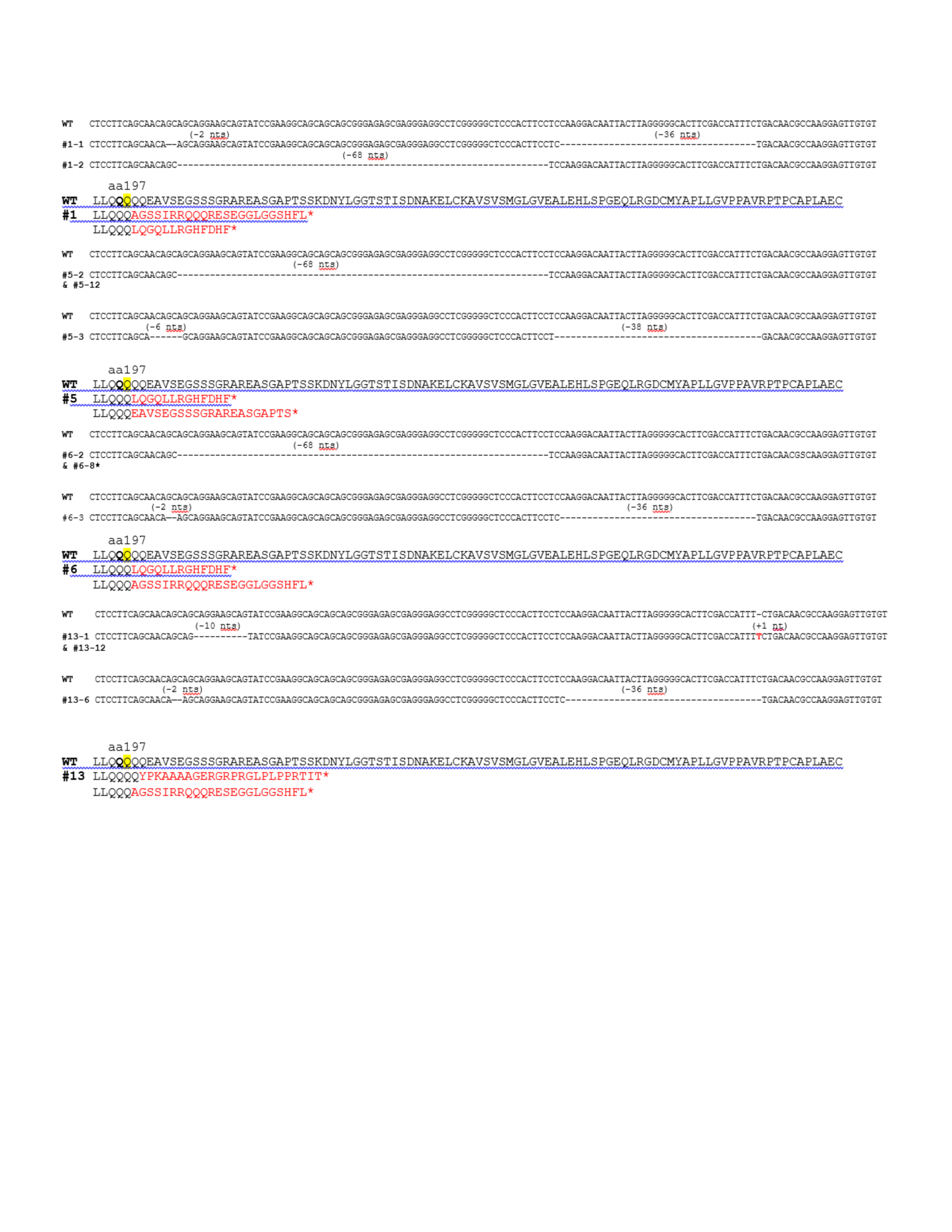

Supplement: Supplementary file 1 [file cancers-17-02535-s001.zip › Figure_S2.tif]

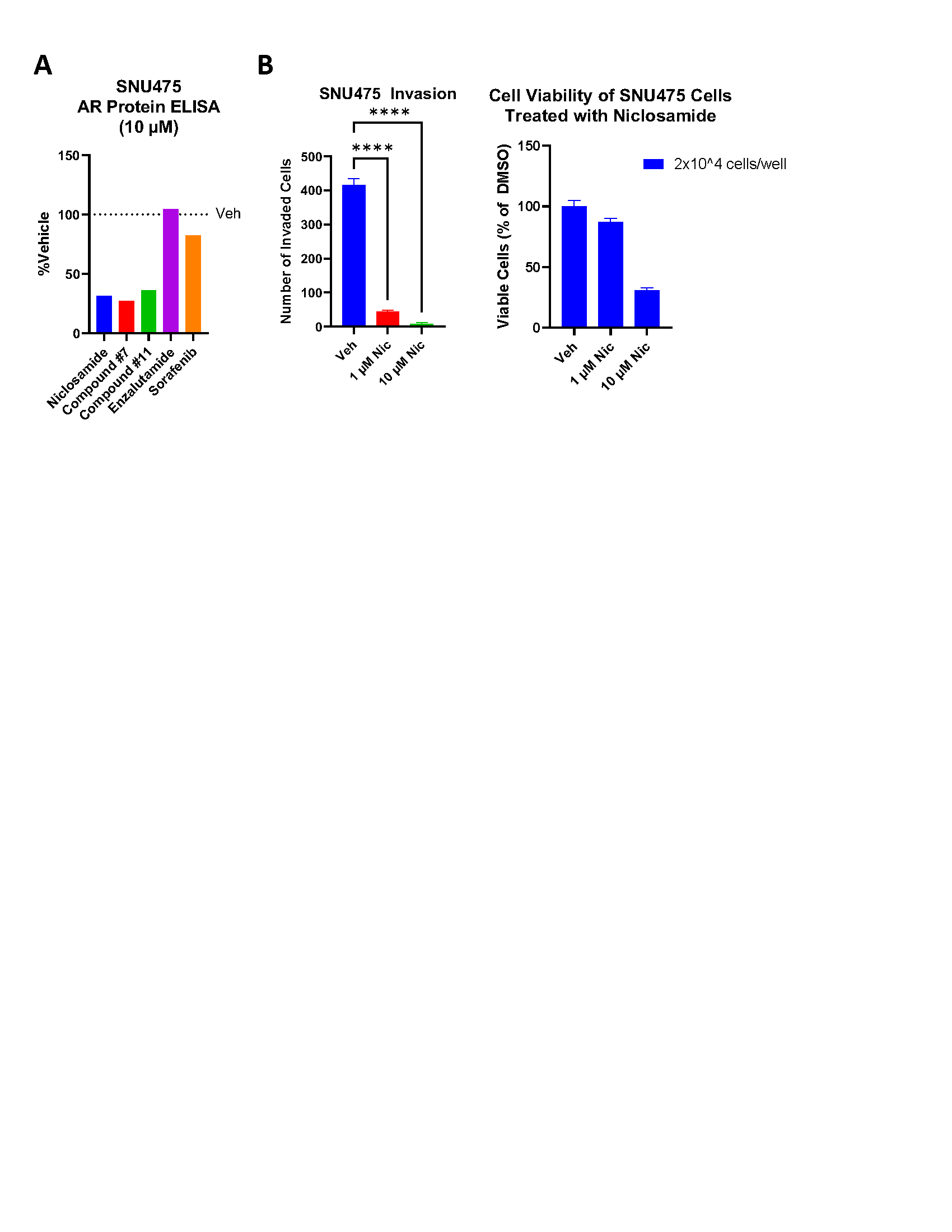

Supplement: Supplementary file 1 [file cancers-17-02535-s001.zip › Figure_S3.tif]

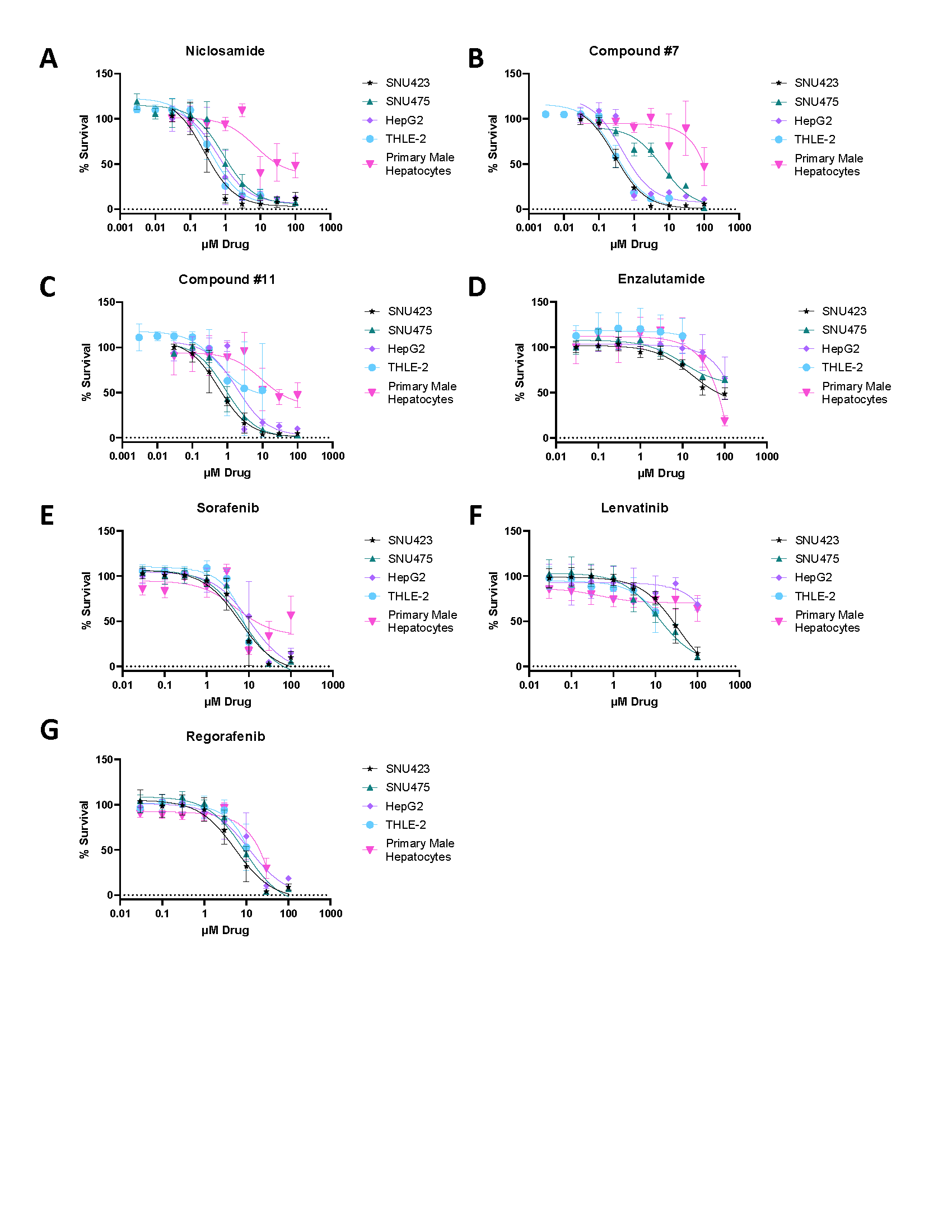

Supplement: Supplementary file 1 [file cancers-17-02535-s001.zip › Figure_S4.tif]

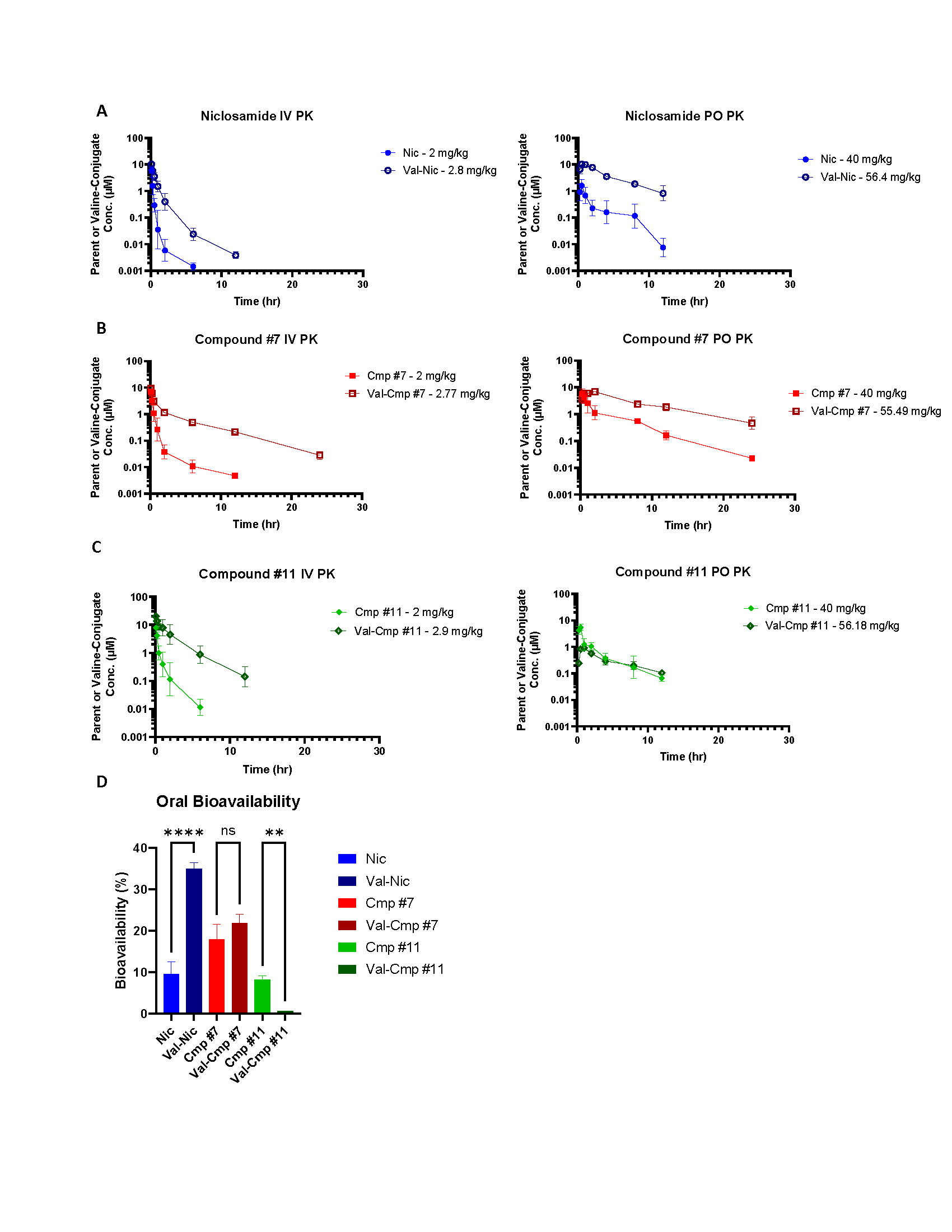

Supplement: Supplementary file 1 [file cancers-17-02535-s001.zip › Figure_S5.tif]

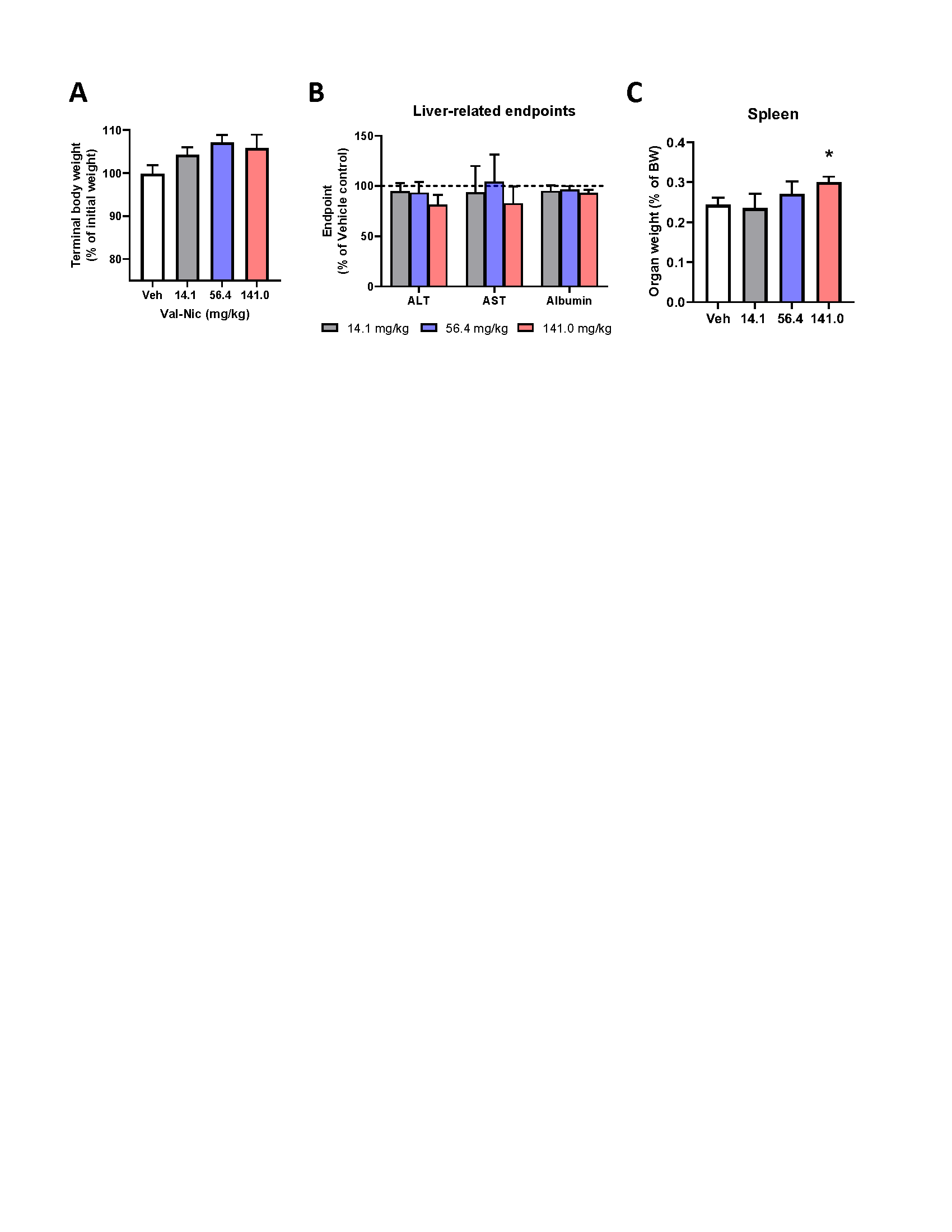

Supplement: Supplementary file 1 [file cancers-17-02535-s001.zip › Figure_S6.tif]

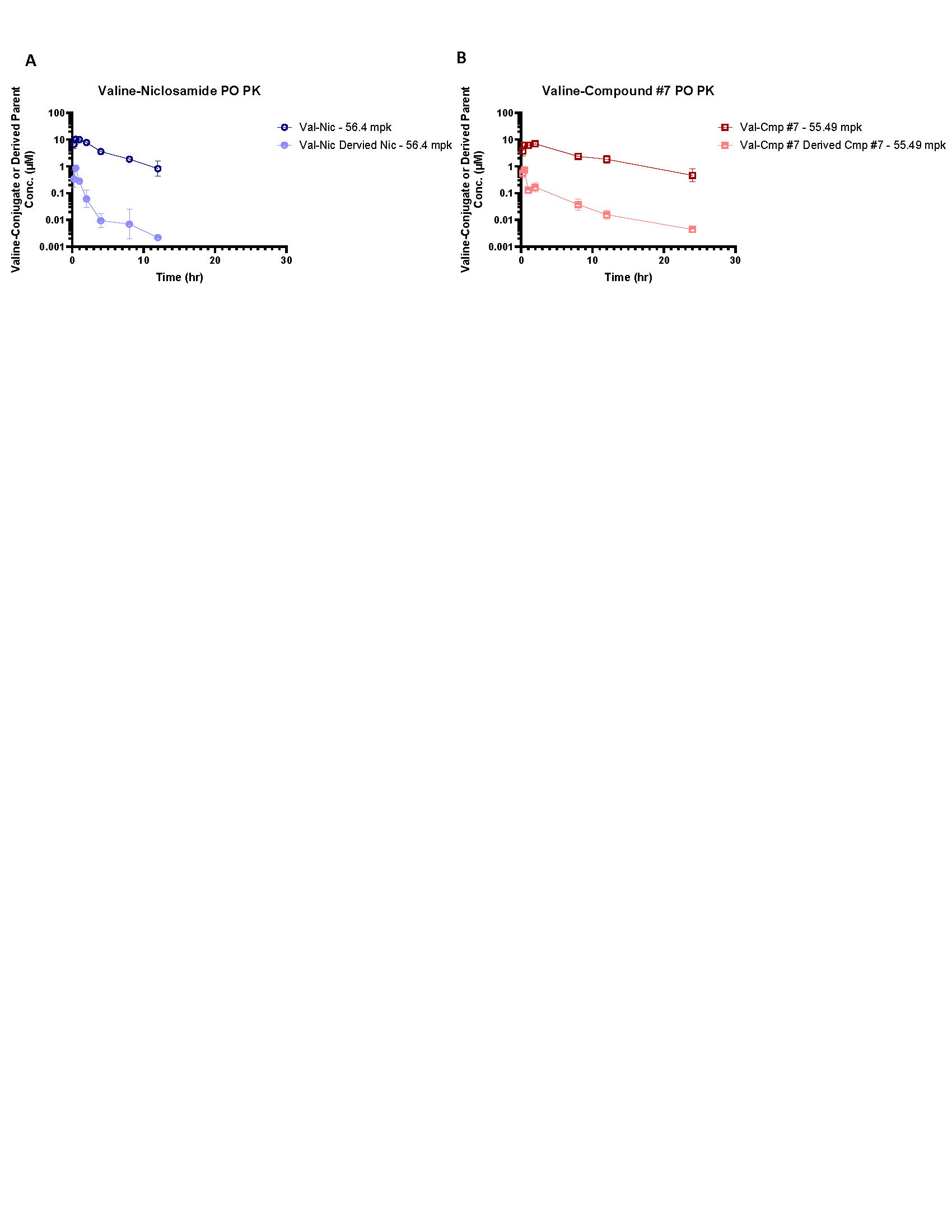

Supplement: Supplementary file 1 [file cancers-17-02535-s001.zip › Figure_S7.tif]

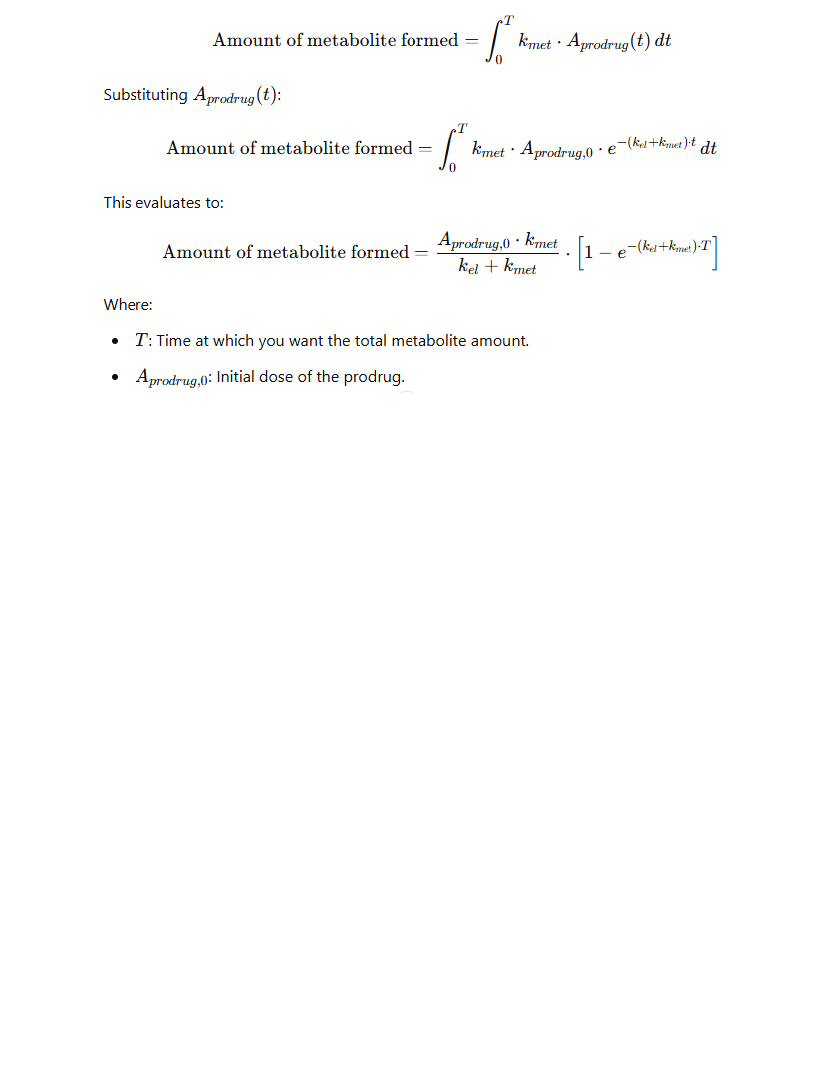

Supplement: Supplementary file 1 [file cancers-17-02535-s001.zip › Figure_S8.tif]

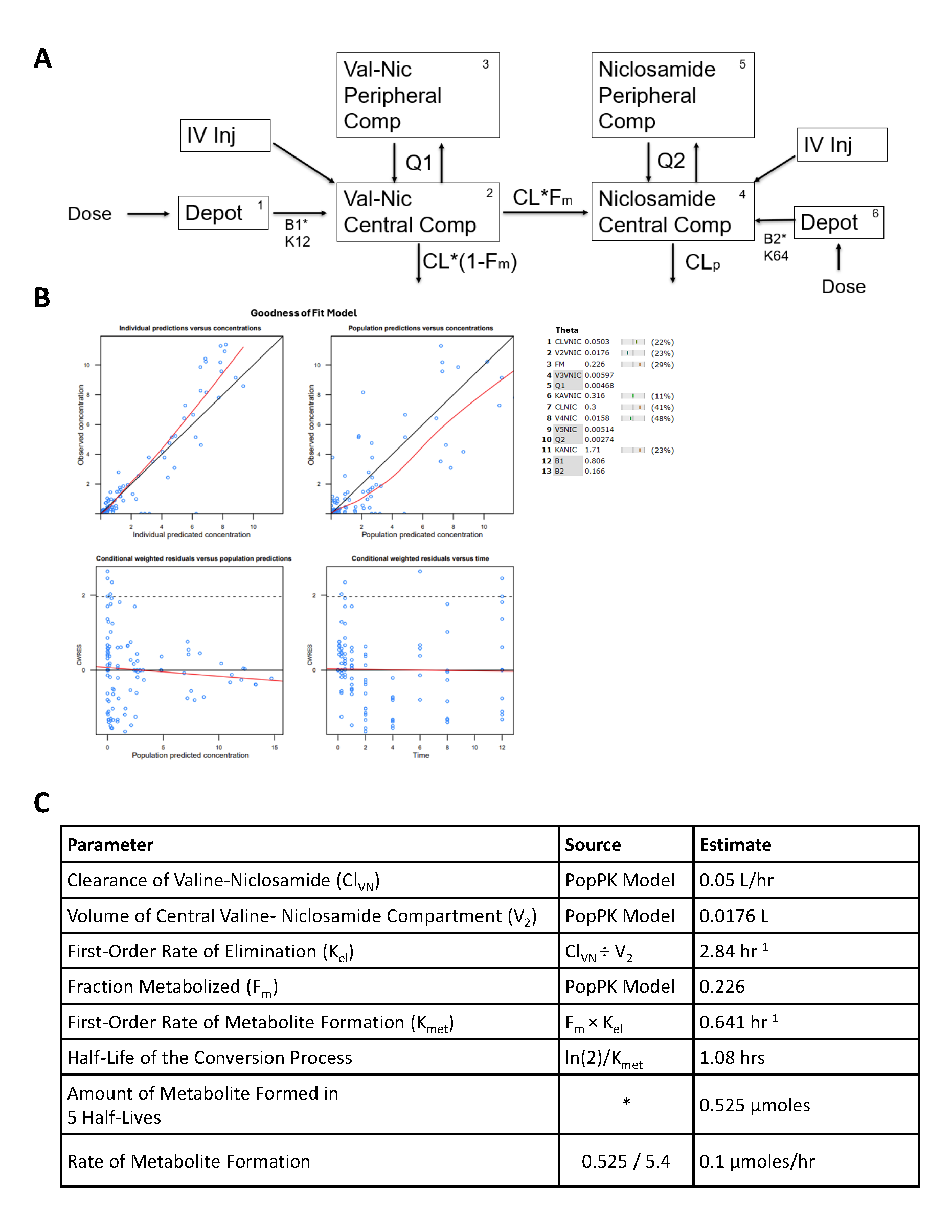

Supplement: Supplementary file 1 [file cancers-17-02535-s001.zip › Figure_S9.tif]
